# Quantifying metabolic energy contributions in sprint running: a novel bioenergetic model

**DOI:** 10.1007/s00421-025-05831-0

**Published:** 2025-06-19

**Authors:** Jérémy Briand, Pietro Enrico di Prampero, Cristian Osgnach, Guy Thibault, Jonathan Tremblay

**Affiliations:** 1 École de kinésiologie et des sciences de l’activité physique (EKSAP), Faculté de médecine, Centre d’éducation physique et des sports (CEPSUM), Université de Montréal, 2100, Boul. Édouard-Montpetit, Montreal, QC H3T 1J4 Canada; 2 Institut de cardiologie de Montréal, Montreal, QC Canada; 3 Department of Sport Science, Exelio Srl, Udine, Italy; 4 Emeritus Professor of Physiology, University of Udine, Udine, Italy

**Keywords:** Bioenergetics, Mathematical modelling, Sprint running, Energy expenditure, Human performance

## Abstract

**Purpose:**

To develop a bioenergetic model representing the dynamics of metabolic power—including aerobic, anaerobic lactic, and anaerobic alactic contributions—during 100–400 m sprints. This study calculates maximum anaerobic capacities using sprint data and assesses the model’s ability to predict performance across various sprint distances.

**Methods:**

Sprint energetics were estimated applying di Prampero et al. (J Exp Biol 208:2809–2816, 2005) method using velocity and time-split data from the 2009 World Athletics Championships to model metabolic power over the men’s and women’s 100–200-400 m events. Aerobic power was modeled with an exponential function, anaerobic lactic power with a bi-exponential function, and anaerobic alactic power with a log-normal function. Maximal anaerobic lactic and alactic capacities were estimated from available performances. Simulations were made to predict the distance traveled by hypothetical male and female athletes achieving World Championship performances on the 100–200-400 m.

**Results:**

The model closely fit metabolic power trajectories (*R*^2^ = 0.94–0.98). Maximal anaerobic alactic capacities were 376 J kg^−1^ (male) and 259 J kg^−1^ (female), and maximal anaerobic lactic capacities were 1314 J kg^−1^ (male) and 1194 J kg^−1^ (female). Simulations of distance traveled revealed mean absolute errors of 0.31% and 1.63% for male and female, respectively. Higher female errors likely stem from underestimating anaerobic lactic contribution due to male-derived parameters and limited available data.

**Conclusion:**

This model aligns closely with theoretical bioenergetic principles and experimental findings, providing valuable insights that improve our understanding of sprint running energetics and performance. Further refinements, incorporating female-specific parameters and collecting data from various distances, could broaden the model’s applicability.

**Supplementary Information:**

The online version contains supplementary material available at 10.1007/s00421-025-05831-0.

## Introduction

World records in athletics represent peak performances achieved by humans at a specific point in time. These performances are often realized in optimized conditions and controlled environment, providing measurements of running time and velocity, comparable to, or even exceeding the precision obtained in controlled laboratory experiments (di Prampero and Osgnach 2018; Hill 1925). The availability of such precise data has, since the early 1900s, sparked interest among scientists studying muscle and exercise physiology (Hill 1925; Kennelly 1926). The analysis of world record performances, from the 100 m to the marathon, has inspired a range of mathematical models that enhance our understanding of the bioenergetics and biomechanics of running (di Prampero et al. 2005; Morton 2006; Péronnet & Thibault 1989; Thongsit et al. 2019). Some of these models have been used to estimate the metabolic power to achieve running performances and the contribution of aerobic and anaerobic energy sources over various running distances, from sprints to marathon (Gastin 2001; Morton 2006; Péronnet & Thibault 1989).

Bioenergetic models by di Prampero et al. (1986; 1993) and Péronnet and Thibault (1989) predict world record performances for distances ranging from 100 m to middle distances (di Prampero et al. 1993) and even the marathon (Péronnet and Thibault 1989). These models show how aerobic and anaerobic energy contributions change with running, while also accounting for the energy required to accelerate from rest to average velocity. In both models, the energy expenditure required for accelerated running was estimated based on changes in the body’s kinetic energy, assuming an efficiency of 0.25. Both models employ an equation from di Prampero et al. (1986, 1993) to calculate the average metabolic power required for running at an average velocity over a given distance. According to this equation, the metabolic cost of acceleration accounts for approximately 36%, 22%, and 10% of the total energy expenditure during 100, 200, and 400 m sprints, respectively, but becomes negligible (less than 4%) for distances of 800 m or more.

Using available data of the time course of running velocity during sprint events (Graubner and Nixdorf 2011; Hernández Gomez et al. 2013; Müller and Hommel 1997), di Prampero et al. (2005; 2018) proposed an alternative approach that relates accelerated or decelerated running on flat terrain to being biomechanically equivalent to uphill or downhill running at a constant velocity. The “Equivalent Slope” in this analogy corresponds to the ratio between the forward acceleration and the acceleration of gravity. This approach yields results like the earlier, simpler equation proposed by di Prampero et al. (1986; 1993) when average metabolic power is considered (di Prampero and Osgnach 2018). However, this updated approach reveals that during the acceleration phase of a sprint, metabolic power peaks at a substantially higher level before progressively declining to a constant value as speed stabilizes (di Prampero et al. 2005, 2023; di Prampero and Osgnach 2018). Applying this method, di Prampero and Osgnach (2018) calculated peak metabolic power for Usain Bolt’s 100 and 200 m world records. They reported peaks of 160 W kg^−1^ and 100 W kg^−1^, respectively, with metabolic power tapering to approximately 65 W kg^−1^ and 50 W kg^−1^ in the later phases of the sprint. Similar findings apply for men’s 400 m performance at the 2009 Athletics World Championships. (di Prampero and Osgnach 2018). These metabolic power peaks presumably reflect significant utilization of anaerobic alactic energy stores in the first seconds of the sprints.

Although di Prampero et al.’s (2005; 2018) method to appreciate the energy expenditure of accelerated running offers new insights on sprint energetics, directly measuring contributions from aerobic, anaerobic lactic and alactic metabolisms contributions to this power production remains challenging. This difficulty is due to the predominant reliance on anaerobic energy sources and the short duration of these events, which prevents the attainment of steady states (di Prampero 1981; di Prampero and Osgnach 2018). Consequently, until now, sprint energetics have primarily been estimated indirectly through biomechanical analyses, assuming an efficiency of metabolic to mechanical energy conversion (Cavagna et al. 1971; Fenn 1930a, 1930b; Kersting 1997; Mero et al. 1992; Murase et al. 1976; Plamondon & Roy 1984). Alternatively, bioenergetics models have provided estimates of aerobic and anaerobic energy contributions (Alvarez-Ramirez 2002; di Prampero et al. 1993; Gastin 2001; Lloyd 1967; Morton 2006; Péronnet and Thibault, 1989; Ward-Smith 1985, 1999). However, only few models distinguish anaerobic lactic and alactic sources (Behncke 1993, 1997; Harman 2002; Heck et al. 2003; Mader 2003; Rodríguez and Mader 2011). Notably, none of the current bioenergetic models incorporate the high metabolic peaks observed around 1 s into sprint running using di Prampero et al.’s (2005; 2018) acceleration cost approach.

This study aims to: 1) Develop a model capturing the time course of metabolic power (aerobic, anaerobic lactic, and anaerobic alactic) in 100–400 sprints, incorporating the energy cost of acceleration as determined by the approach introduced by di Prampero et al. (2015, 2005, 2023; 2018); 2) Estimate maximum anaerobic lactic and alactic capacities from sprint performance data; 3) Interpret and discuss the predictions and practical application of this sprint running energetics model considering available theoretical knowledge and experimental data.

## The energy cost of sprint running

Accelerating demands more energy per meter than maintaining a constant velocity. Research by di Prampero et al. (2015, 2005, 2023; 2018) indicates that running with forward acceleration on flat terrain is energetically comparable to running uphill at a steady pace up an “Equivalent Slope” mentioned above. For more details, readers can consult prior studies (di Prampero et al. 2015, 2005, 2023; di Prampero and Osgnach 2018; Osgnach et al. 2023). Since the energy cost of uphill running has been studied for decades (Margaria 1938; Minetti et al. 2002), this relationship allows for the estimation of the energy cost in accelerated running. From this, the instantaneous metabolic power (W kg^−1^) can be calculated by multiplying this energy cost (J kg ^−1^ m^−1^) by velocity (m s^−1^), knowing the time course of velocity and acceleration (m s^−2^) during a race.

### Estimating metabolic power

We computed the time course of metabolic power in men’s and women’s 100, 200, and 400 m sprints using velocity and time-split data from Graubner and Nixdorf (2011). The data originated from the sprint events of the 2009 World Athletics Championships in Berlin. Velocity and time splits were measured at 10 m intervals for men’s 100 m, 20 m for women’s 100 m, and 50 m for both 200 and 400 m races.

Following studies applying the uphill equivalence method to estimate the energy cost of accelerated running (di Prampero et al. 2015, 2005; di Prampero and Osgnach 2018; Morin et al. 2019), velocity during the acceleration phase was assumed to increase exponentially, as in the following equation:

1 $$v\left(t\right)={v}_{f}\bullet \left(1-{e}^{\frac{-t}{\tau }}\right)$$

where $v(t)$ represents the instantaneous velocity (m s^−1^) at time $t$ (s), ${v}_{f}$ is peak velocity (m s^−1^), and $\tau$ (s) is the time constant of the velocity exponential rise. Table 1 presents ${v}_{f}$ and $\tau$ values for men’s and women’s 100, 200, and 400 m races.

**Table 1:** Peak velocity (${\text{v}}_{\text{f}}$, m s^−1^) and time constants ($\uptau$, s) for distances of 100, 200 and 400 m, derived from performances at the 2009 World Athletics Championships in Berlin

Distance (m)	Athlete	$\tau$(s)	${v}_{f}$(m s^−1^)
Men			
100	Bolt	1.27	12.34
200	Bolt	1.59	11.57
400	Merritt	1.54	9.88
Women			
100	Fraser-Pryce	1.34	10.58
200	Felix	1.59	10.18
400	Williams	1.52	9.12

These include Bolt’s 100 m (9.58 s) and 200 m (19.19 s), Merritt’s 400 m (44.06 s), Fraser-Pryce’s 100 m (10.73 s), Felix’s 200 m (22.02 s), and Williams’ 400 m (49.32 s)

To estimate $\tau$, we fitted Eq. 1 to the average velocity data using non-linear least squares regression, employing the Levenberg-Marquardt algorithm as implemented in the *minpack.lm* R package (Elzhov et al. 2023). Average velocities were computed for intervals determined by the time splits data (e.g., every 10, 20, or 50 m) and were associated with a running time corresponding to the midpoints of those intervals. Reaction time was subtracted from the time measurements prior to performing the fitting process.

Data from Graubner and Nixdorf (2011) used for the current analysis cover Bolt’s 100 m (9.58 s) and 200 m (19.19 s), Merritt’s 400 m (44.06 s), Fraser-Pryce’s 100 m (10.73 s), Felix’s 200 m (22.02 s), and Williams’ 400 m (49.32 s). All represent first-place finishes, except Williams’ second-place 400 m. We excluded data from Richards, the women’s 400 m winner, due to a marked slowdown in her second 100 m (Graubner and Nixdorf 2011), invalidating post-acceleration phase constant-velocity assumption. Video footage, referred to in the supplementary material, suggests Richards accelerated early to benefit from Williams’ aerodynamic draft on the backstretch.

Non-linear least-squares regression performance for determining $\tau$ were assessed using root mean square error (RMSE) and residual standard error (RSE). For the 100 m sprints, the model exhibited a strong fit, with an RSE of 0.13 m s⁻^1^ for the men’s data and 0.24 m s⁻^1^ for the women’s data, and an RMSE of 0.12 m s⁻^1^ and 0.20 m s⁻^1^ for the men’s and women’s data, respectively. Model assumptions were validated through residual analysis. Homoscedasticity was evaluated by plotting residuals against fitted values, the normality of residuals was reviewed using Q–Q plots, and systematic deviations were examined visually via residual plots. Minor deviations from normality in the residuals were observed during the late phase of the 100 m sprints, which is where slight deceleration typically occurs in the race. These minor discrepancies were anticipated, as the model described in Eq. 1 does not explicitly account for the deceleration phase observed in the sprint instantaneous velocity profile.

For the 200 m and 400 m sprints, the coarser resolution of the split data (measured at 50 m intervals) necessitated extending the fitting range up to 200 m to ensure a sufficient number of data points for the non-linear least-squares fitting procedure. This adjustment increased the influence of the deceleration phase on the fit, leading to higher residual errors during that phase. The RSE values were 0.36, 0.44, 0.12, and 0.25 for the men’s 200 m, women’s 200 m, men’s 400 m, and women’s 400 m events, respectively. These larger errors were particularly evident in the greater deviation of residuals from normality observed during the deceleration phases.

Including velocities during the deceleration phase in the non-linear least-squares fitting approach results in an underestimation of ${v}_{f}$. Consequently, values of ${v}_{f}$ were adjusted based on the highest instantaneous velocities reported by Graubner and Nixdorf (2011) for 100 m races or by using the highest average velocities over the available time intervals, for the 200 and 400 m races. While this adjustment has minimal impact on the 100 m fits, it improves the residual patterns in the acceleration phases of the 200 m and 400 m races. The values of $\tau$ and ${v}_{f}$ derived using the described approach are presented in Table 1. For the men’s event, the value closely aligns with the parameters $\tau$ and ${v}_{f}$ estimated by di Prampero and Osgnach (2018) using the same data, with only minor differences attributed to our inclusion of reaction time.

We calculated the time at which peak velocity is reached, marking the acceleration phase’s end, as the midpoint of the interval with the highest average velocity. We calculated instantaneous acceleration $a\left(t\right)$ (m s^−2^) in the acceleration phase with the following equation:

2 $$a\left(t\right)=\frac{{v}_{f}}{\tau }\bullet {e}^{\frac{-t}{\tau }}$$

**Fig. 1:**
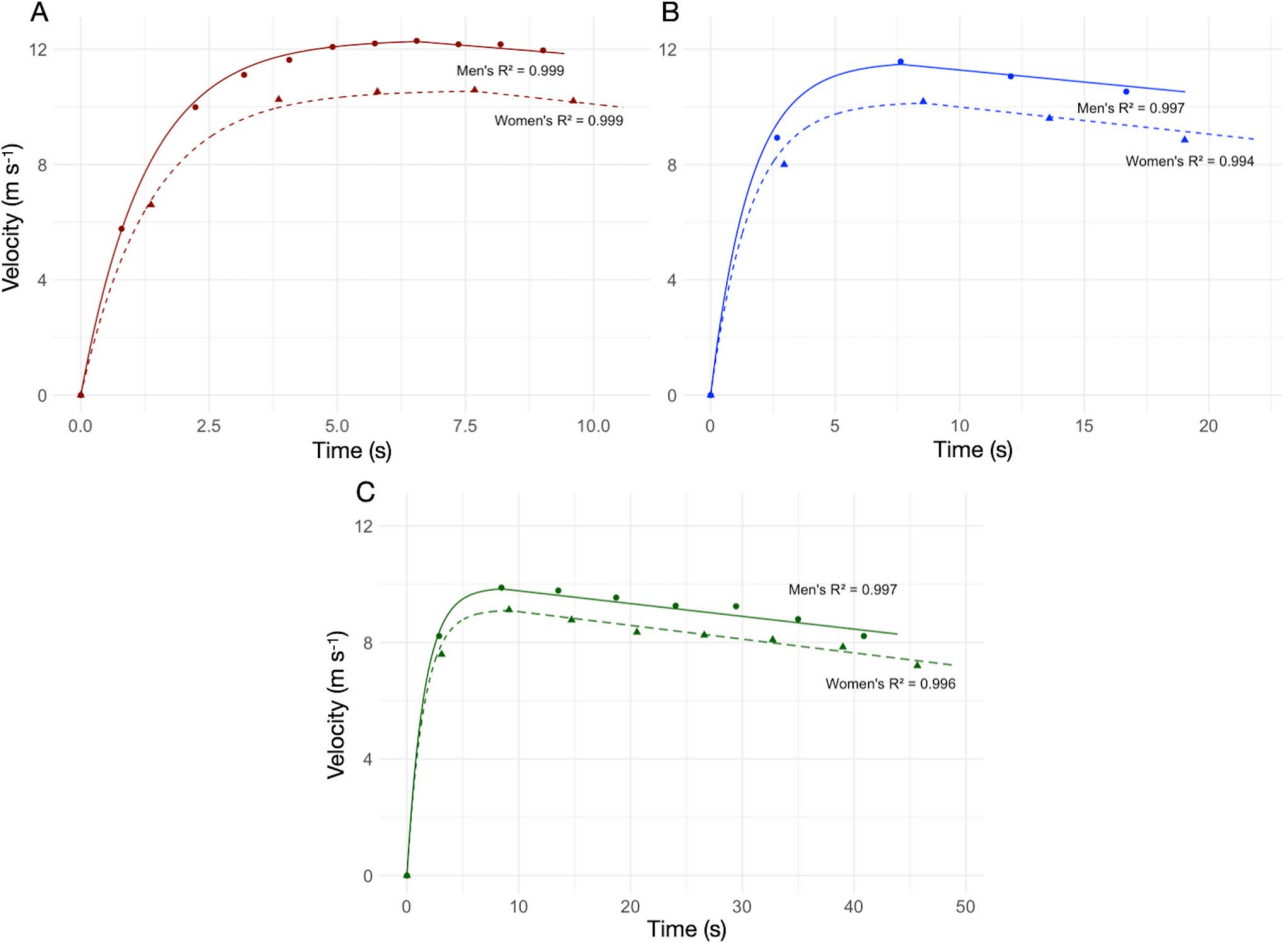
Velocity time course during 100 m (**A**), 200 m (**B**), and 400 m (**C**). Solid points show measured velocities from Graubner and Nixdorf (2011): circle for men, triangles for women. Lines represent modeled velocities from Eq. 1 and linear interpolation post-${\text{v}}_{\text{f}}$. Solid lines denote men’s data, dashed lines women’s. *R*^2^ values reflect model goodness of fit. Integration of modeled velocity over time gives distances of 99.93 m (men) and 96.84 m (women) for 100 m, 195.69 m (men) and 197.31 m (women) for 200 m, and 389.49 m (men) and 395.54 m (women) for 400 m

To better represent the instantaneous velocity profile over the whole sprint distance, as in di Prampero and Osgnach (2018)’s previous work, we assumed that runners decelerate linearly after reaching peak velocity for the remainder of the race. We derived the races’ final velocity by linearly interpolating the average velocity during the last time interval, using the finishing time and the remaining distance to be covered at that point. We modeled the deceleration phase’s velocity time course by linear interpolation between ${v}_{f}$ and final velocity. Figure 1 presents a comparison between measured velocities and modeled instantaneous velocity for 100 m, 200 m, and 400 m sprints in both men and women. During the acceleration phase of the sprint, velocity estimations were derived using Eq. 1 and the constants provided in Table 1. The RSE and RMSE values of the instantaneous velocity model, compared to the velocities reported by Graubner and Nixdorf (2011), are detailed in the supplementary materials.

We used instantaneous acceleration and velocity to estimate running energy cost and metabolic power (calculated as the product of energy cost and velocity) at each time point. Energy cost derivation from acceleration and velocity is described in further detail in di Prampero and Osgnach (2018). Beyond the attainment of peak velocity, after the acceleration phase, we assumed an energy cost of 3.8 J kg^−1^·m^−1^, consistent with flat-terrain running (di Prampero and Osgnach 2018; Minetti et al. 2002). This assumption can be justified by the fact that runners in the deceleration phase slow down due to an inability to sustain energy output, rather than by deliberate choice. Since their posture and center of mass remain stable during this phase, the flat-terrain energy cost calculation remains applicable.

**Fig. 2:**
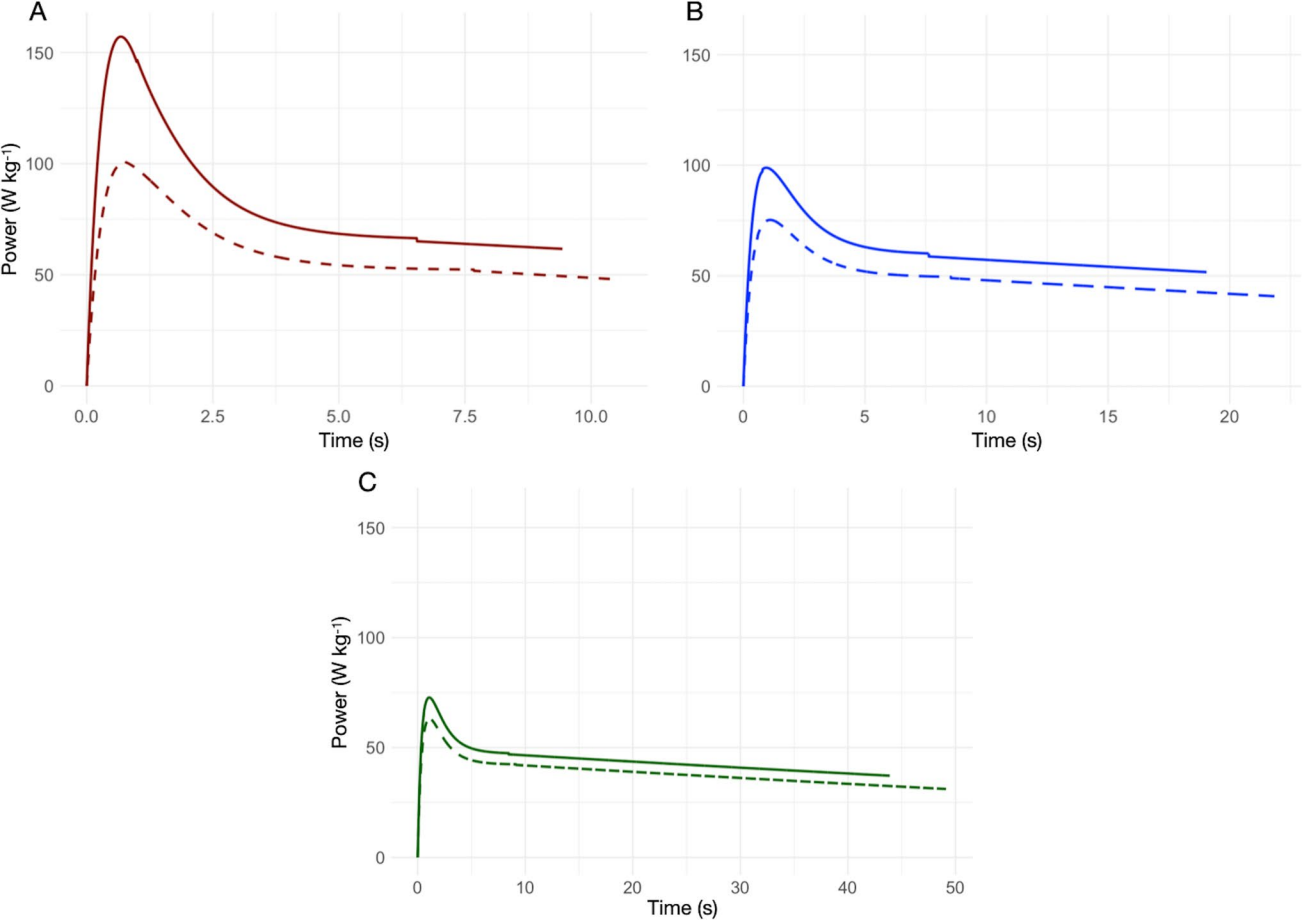
Metabolic power as a function of running time derived using di Prampero et al. methods (2005; 2018). Solid lines depict men’s performances, dashed lines women’s. Panels A-C display metabolic power for 100 m, 200 and 400 m sprints, respectively: **A** 100 m-men:total energy 772 J kg^−1^, average power 82.9 W kg^−1^, peak power 157 W kg^−1^, acceleration cost 589 J kg^−1^; women: total energy 638 J kg^−1^, average power 60.3 W kg^−1^, peak power 101 W kg^−1^, acceleration cost 494 J kg^−1^; **B** 200 m-men: total energy 1172 J kg^−1^, average power 61.5 W kg^−1^, peak power 99 W kg^−1^, acceleration cost 543 J kg^−1^; women: total energy 1075 J kg^−1^, average power 49.2 W kg^−1^, peak power 75 W kg^−1^, acceleration cost 479 J kg^−1^; **C** 400 m-men: total energy 1942 J kg^−1^, average power 44.3 W kg^−1^, peak power 73 W kg^−1^, acceleration cost 456 J kg^−1^; women: total energy 1459 J kg^−1^, average power 38.5 W kg^−1^, peak power 63 W kg^−1^, acceleration cost 431 J kg^−1^. Power discontinuities near 6 s reflect linear deceleration post-acceleration (see text)

We adjusted the energy cost for the air resistance (J kg^−1^ m^−1^) by adding, to the previously calculated energy cost, 0.01 *v*(*t*), where $v\left(t\right)$ is instantaneous velocity (di Prampero 1986; Pugh 1970). Figure 2 depicts the metabolic power time course for men’s and women’s 100, 200, and 400 m sprints The Fig. 2 caption details peak and average metabolic power, total energy, and energy of the acceleration phases for men and women performances. Having mapped metabolic power in sprint events, the next step is to analyze energy contributions from anaerobic alactic, lactic, and aerobic systems to construct a comprehensive sprint energy model.

## Aerobic and anaerobic energy contributions

Total running energy expenditure combines contributions from aerobic and anaerobic systems (di Prampero 1981). Anaerobic energy stems from two sources: alactic metabolism (breakdown of ATP and PCr) and lactic (or glycolytic) metabolism. The latter is typically evaluated through various methods, including assessing total accumulated blood lactate during exercise (di Prampero 1981). To evaluate the contributions of aerobic, anaerobic lactic, and anaerobic alactic metabolisms to the total energy expenditure in sprint running, we made several assumptions based on theoretical principles.

### Aerobic energy contribution

We modeled the time course of the aerobic power contribution (${P}_{aer\left(t\right)}$, in W kg^−1^) as an exponential rise to maximal aerobic power (MAP) (di Prampero et al. 1993; Péronnet and Thibault 1989), with a time constant (${k}_{aer}$) of 23 s (Capelli et al. 2001; Cautero et al. 2002; di Prampero et al. 2005). The MAP includes the basal metabolic energy rate (BMR), which we consider fixed at 1.2 W kg^−1^, equivalent to an oxygen consumption of 3.5 mL O_2_ kg^−1^ min^−1^ (Péronnet and Thibault 1989). Our analysis focuses on aerobic energy expended above BMR.

3 $${P}_{aer}\left(\text{t}\right)=\left(MAP-BMR\right)\bullet \left(1-{e}^{\frac{-t}{{k}_{aer}}}\right)$$

Based on the maximal oxygen consumption (VO_2_max) values of Spanish runners reported by Legaz-Arrese et al. (2007), the highest VO_2_max measured for men and women sprint runners are approximately 70 mL O_2_ kg⁻^1^ min⁻^1^ and 60 mL O_2_ kg⁻^1^ min⁻^1^, respectively. Using the energy equivalent of 20.9 J/mL O_2_, we estimated a MAP of 24.5 W/kg for men and 21 W/kg for women.

Available experimental data on elite sprinters indicate that the highest VO_2_max values measured are approximately 70 mL O_2_·kg⁻^1^·min⁻^1^ for men and 60 mL O_2_·kg⁻^1^·min⁻^1^ for women (Legaz-Arrese et al. 2007; Saltin and Åstrand 1967). Although the sprinters whose performances were analyzed in the current study come from different countries than those in the original studies reporting VO_2_max values in elite sprinters, research has shown that VO_2_max values are not affected by ethnicity (Hunter 2017). Using the energy equivalent of 20.9 J mL^−1^ O_2_, we therefore estimated the MAP to be 24.5 W kg^−1^ for male elite sprinters and 21 W kg^−1^ for female elite sprinters.

### Anaerobic alactic energy contribution

At rest, muscle ATP and PCr concentrations are typically around 6 mmol kg^−1^ and 20 mmol kg^−1^ of fresh muscle tissue, respectively (Francescato et al. 2008, 2003). For elite male athletes performing at world-record velocities, we estimate that maximally active muscles comprise ~ 20–25% of body mass. To maintain sufficient ATP levels and prevent declines that could impair muscle power output (di Prampero and Piiper 2003), we assume only a total of about 20 mmol kg^−1^ of PCr per kg of active muscle mass can be utilized from rest to exhaustion. This corresponds to an estimated 4–5 mmol of PCr per kg of body mass. Assuming a P/O ratio of 6 (mol/mol), we can calculate that ~ 0.67–0.83 mmol O₂ kg^−1^ of body mass is conserved from PCr splitting. Given the energy equivalent of 20.9 J mL^−1^ O₂ and the fact that 1 mmol O₂ equals 22.4 mL, we estimate the maximum energy released from PCr splitting ranges between 310 and 390 J kg^−1^ of body mass, for male athletes (di Prampero and Osgnach 2018).

Since females’ skeletal muscle proportion is ~ 75% of males’ (Janssen et al. 2000; Miller et al. 2024), we can assume that the maximally active muscles in female athletes constitute approximately 15–20% of their body mass. Using the same approach as for men, we estimate the maximum energy released from PCr splitting ranges between 230–292 J kg^−1^ of body mass, for female athletes.

### Anaerobic lactic energy contribution

Maximal 400 m sprints typically elevate blood lactate concentrations by 15–20 mmol L^−1^ above resting levels (Arcelli et al. 2014; Hanon et al. 2010; Hautier et al. 1994) though higher values up to 25 mmol L^−1^ have been observed and documented (Heck et al. 2003; Lacour et al. 1990). The accumulation of 1 mmol L^−1^ of blood lactate corresponds to an energy expenditure from the anaerobic lactic metabolism equivalent to the consumption of 3 mL O₂ kg^−1^ of body mass (di Prampero 1981; di Prampero and Ferretti 1999). Thus, a blood lactate accumulation of 15–20 mmol L^−1^ reflects an energy release from the anaerobic lactic metabolism ranging from 940 to 1250 J kg^−1^ (di Prampero and Osgnach 2018).

Although lactic power contribution is lower in women sprinters than in men (Ward-Smith and Radford 2000), peak lactate levels in sprints up to 800 m show no significant sex difference (Korhonen et al. 2003; Lacour et al. 1990). This can be explained in part by differences in body composition and lactate space between male and female athletes (Taylor and Péronnet 1981). To clarify this, if we consider the energy equivalent of 20.9 J mL^−1^ O₂, the consumption of 3 mL O₂ kg^−1^ of body mass generates 62.7 J kg^−1^ of anaerobic lactic energy per kg of body mass. Since 162 g of glycogen (1 glycosyl unit) provides 178 g of lactate, releasing 47 kcal, equivalent to 197 kJ of energy, we can calculate the energy equivalent of lactate to be 1109 J g^−1^ (197 J ÷ 178 g = 1.109 J g^−1^) (Stryer 1995). Therefore, the production of 62.7 J kg^−1^ corresponds to the accumulation of 0.0568 g of lactate per kilogram of body mass. With a lactate molar mass of 89 g mol^−1^ and assuming a homogenous distribution of lactate throughout the body (di Prampero 1981), a lactate space of 63.8% would be required. With lean tissues composed of 73% water (Wang et al. 1999), an accumulation of 1 mmol L^−1^ yielding 3 mL O₂ per kilogram of body mass is reasonable for individuals with approximately 12.5% body fat, which is a reasonable value for male elite sprinters (Perez-Gomez et al. 2008).

Female sprinters, however, typically exhibit higher body fat percentage than male (Miller et al. 2024; Perez-Gomez et al. 2008). If we consider, for female athletes, that 1 mmol L^−1^ of lactate corresponds to an energy expenditure equivalent to the consumption of 2.7 mL O₂ kg^−1^ of body mass (di Prampero 1981; di Prampero and Ferretti 1999), then, to reach an energy output of 56.7 J kg^−1^ of body mass, a lactate space of 57% would be necessary. This is reasonable for individuals with around 20% body fat and aligns with the body composition observed in elite female athletes (Miller et al. 2024). Thus, for elite female sprinters, a blood lactate accumulation of 15–20 mmol L^−1^ results in an energy release from the anaerobic lactic metabolism ranging approximately 850–1130 J kg^−1^.

## Approximate metabolic power distribution

Based on previous theoretical estimates, we began by approximating the distribution of aerobic, anaerobic lactic, and alactic power in sprint running to guide our bioenergetic model development. We based this approximation on the metabolic power time course depicted in Fig. 2. We initially analyzed the men’s 100 m sprint, before extending the approach to both men’s and women’s 200 and 400 m. Alactic power dominates the sprint’s initial seconds (Graubner and Nixdorf 2011). We thus evaluated approximate metabolic contributions by separately analyzing the acceleration and deceleration phases of the men’s 100 m event.

### Acceleration phase

We calculated the energy expended during the acceleration phase of the men’s 100 m sprint using numerical integration (see caption Fig. 2). Since the calculated energy expenditure of 589 J kg^−1^ is larger than the estimated maximal anaerobic alactic capacity for an elite male athlete, the energy expended in the acceleration phase cannot be solely attributed to the anaerobic alactic metabolism.

Aerobic metabolism contributes minimally to a 100 m sprint (di Prampero 1981). With Eq. 3 and a MAP of 24.5 W kg^−1^ (estimated for male elite sprinters), we calculated the aerobic contribution during the acceleration phase, which roughly corresponds to the first 6 s of the 100 m sprint. Integrating ${P}_{aer}\left(\text{t}\right)$ over time yields an aerobic energy contribution of 20 J kg^−1^ during the acceleration phase. Subtracting this aerobic contribution from the total power of the acceleration phase gives ~ 570 J kg^−1^. This value still exceeds the theoretical estimates of the maximum energy provided by PCr and ATP splitting (310 and 390 J/kg). We deduced that the anaerobic energy during the acceleration phase (~ 570 J kg^−1^) can be divided into lactic and alactic sources. Prior bioenergetics models e.g., Ward-Smith, (1985, 1999), Harman, (2002), suggest an exponential rise in lactic power contribution. Gastin (2001) confirms this exponential rise, noting maximal lactic power after ~ 5 s based on experimental data. We therefore consider that the anaerobic lactic power during the acceleration phase (${P}_{an,accel}$, in W kg^−1^) increases exponentially, with a short half-life time constant (${k}_{an}$) of 2 s, peaking at ${\text{P}}_{an,max}$ (W kg^−1^) around the end of the sprint acceleration (~ 6 s).

4 $${P}_{an,accel}\left(\text{t}\right)={\text{P}}_{an,max}\bullet \left(1-{e}^{\frac{-t}{{k}_{an}}}\right)$$

**Fig. 3:**
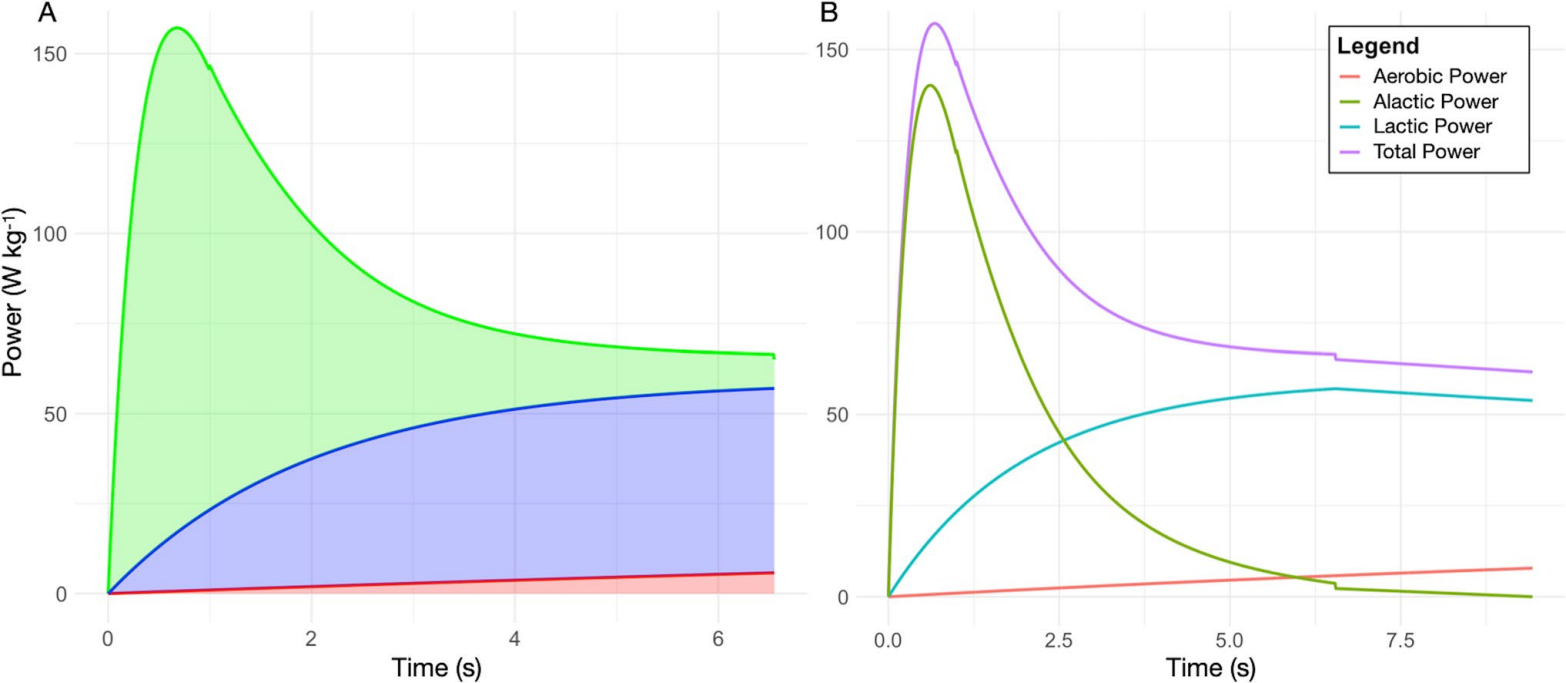
Panel A depicts metabolic power during the 100 m sprint’s acceleration phase. Green, blue and red areas denote alactic (295 J kg^−1^), lactic (274 J kg^−1^), and aerobic (20 J kg^−1^) energy contributions, respectively. Panel B displays approximate power contributions throughout the 100 m sprint. The purple line reflects total metabolic power, modeled per di Prampero et al. (2005; 2018) with 2009 Athletics World Championships data (Graubner and Nixdorf 2011). Discontinuities arise from the modeled shift between acceleration and deceleration phases (~ 6 s, see text)

We finally calculated the energy expended using anaerobic alactic sources by subtracting the integral of ${P}_{an,accel}\left(\text{t}\right)$ over the acceleration phase to the total energy attributed to anaerobic sources (~ 570 J kg^−1^). Figure 3 illustrates the aerobic, anaerobic lactic, and alactic power contributions during 100 m sprint’s acceleration phase. The approximate estimation of the energy contribution from anaerobic lactic metabolism is 274 J kg^−1^, while the energy derived from anaerobic alactic metabolism is about 295 J kg^−1^. Considering the maximal alactic capacity ranging from 310 to 390 J kg^−1^, this alactic energy expenditure of 295 J kg^−1^ over the acceleration of the 100 m race aligns with theoretical expectations and can be attributed to the breakdown of PCr and ATP.

### Deceleration phase

Following the acceleration phase, in each sprint performance, a slight decline in running velocity is observed (Fig. 1). In the men’s 100 m sprint, this deceleration typically occurs over approximately 3 s. During this phase, the energy contribution from anaerobic alactic metabolism becomes marginal, decreasing linearly as the exercise progresses to the point where its power contribution is negligible by the end of the race. In contrast, aerobic metabolism continues to increase exponentially. The remaining and predominate energy source in this phase of the 100 m race comes from anaerobic lactic metabolism. From these considerations, we can estimate the approximate contributions of anaerobic alactic, lactic, and aerobic metabolisms throughout the 100 m sprint, as depicted in Fig. 3.

## Modelling the metabolic power distribution

Using the approximate metabolic power distributions from the men’s 100 m sprint (Fig. 3), we modeled the time course of aerobic, anaerobic lactic, and alactic energy systems in sprint running. Aerobic power contribution follows Eq. 3. Subsequent subsections model the lactic and alactic energy systems.

### Modelling anaerobic lactic power contribution

Ward-Smith (Ward-Smith 1985, 1999), Álvarez-Ramírez (2002), and Harman (2002), model lactic power contribution (${P}_{la}$) during a sprint of duration $T$ (in s) using a bi-exponential function at time $t$ (in s).

5 $${P}_{la}(t)={P}_{la,max}(T){\bullet {k}_{norm}\bullet (1-e}^{\frac{-t}{{k}_{1}}})\bullet {e}^{\frac{-t}{{k}_{2}}}$$

where ${P}_{la,max}\left(T\right)$ is the peak anaerobic lactic power over the race duration (in W kg^−1^) and ${k}_{norm}$ is a normalization constant that depends exclusively on the values of ${k}_{1}$ and ${k}_{2}$, such that ${P}_{la,max}\left(T\right)$ is the maximum of the function. Details on the derivation of ${k}_{norm}$ are provided in supplementary material. The time constants ${k}_{1}$ and ${k}_{2}$ guide the exponential rise and decay of the anaerobic lactic power contribution over the course of a sprint event.

Equation 5 aligns with experimental observations on the time course of the lactic energy contribution during sprints (Gastin 2001; Gastin et al. 1995; Medbo et al. 1988). Lactic power relies on glycolysis, driven by rising AMP, ADP, and inorganic phosphate levels (di Prampero 1981; Heck et al. 2003; Mader 2003). During the acceleration phase, the breakdown of PCr results in elevated ADP levels, triggering an exponential rise in lactic power contribution. Thus, the time constant ${k}_{1}$ is short, typically ranging from 2 to 3 s, causing glycolytic power to peak at approximately 6 s (Gastin 2001). This peak coincides with the transition of the sprint from the acceleration phase to the deceleration phase (Gastin 2001; Heck et al. 2003).

Throughout the sprint, the glycolysis becomes less efficient and is gradually inhibited (di Prampero 1981; Girard et al. 2011; Hargreaves and Spriet 2020; Heck et al. 2003; Mader 2003). This inhibition causes an exponential decay in the glycolytic power contribution. The time constant of this decay can be estimated to be around 30 s (Harman 2002; Gastin 2001). Glycolysis inhibition provides a plausible explanation for the inability to sustain maximal velocity after the acceleration phase of the sprint. We computed total lactic energy by integrating ${P}_{la}\left(t\right)$ over sprint duration $T$.

The amplitude ${P}_{la,max}\left(T\right)$ can be assimilated to the maximal lactic power (di Prampero 1981; Heck et al. 2003) and depends on ADP levels and the energy derived from PCr splitting during the acceleration phase (di Prampero 1981, Mader 2003). As we will show in later sections, ${P}_{la,max}\left(T\right)$ will vary depending on total sprint duration and magnitude of the acceleration peak power.

### Modeling anaerobic alactic power contribution

Anaerobic alactic power contribution exhibits an exponential rise, followed by a slower exponential decay. This pattern, often resembling a skewed normal distribution, is commonly referred to as a log-normal distribution (Limpert et al. 2001). In natural systems, log-normal distributions describe numerous chemical reactions and processes with similar dynamics, especially those constrained by finite enzymatic or metabolic capacities. Many enzymatic activities exhibit a rapid increase in reaction rates as substrate concentrations rise, reaching a peak before levelling off due to substrate depletion or feedback inhibition mechanisms (Limpert et al. 2001). This dynamic mirrors the rise-and-decay shape typical of anaerobic alactic energy release (di Prampero 1981). In a sprint of duration $T$ (in s), the anaerobic alactic power contribution (${P}_{al}$, in W kg^−1^) at any time $t$ (in s) can be computed with the following log–normal function:

6 $${P}_{al}\left(t\right)={P}_{al,max}\left(T\right){\bullet e}^{\left(\frac{{-\left(\text{ln}\left(t\right)- {\mu }_{al}\right)}^{2}}{{2{\sigma }_{al}}^{2}}\right)}$$

where ${P}_{al,max}\left(T\right)$ denotes peak alactic power over duration $T$. ${\mu }_{al}$ is a constant that sets the time at which ${P}_{al,max}\left(T\right)$ is achieved, while ${\sigma }_{al}$ is a constant controlling the function’s decay. We determined total alactic energy by integrating ${P}_{al}\left(t\right)$ over sprint duration $T.$

### Fitting the model to approximate contributions

Total metabolic power in sprint running ${P}_{tot}\left(t\right)$ (in W kg^−1^) combines alactic, lactic, and aerobic power contributions.

7 $${P}_{tot}\left(t\right)= {P}_{al}\left(t\right)+ {P}_{la}\left(t\right)+ {P}_{aer}\left(\text{t}\right)$$

We calculated each energy system’s contribution (in J kg^−1^) by integrating its power over sprint duration, $T$.

After analyzing the men's 100 m, we calculated approximate energy system contributions for the women's 100 m sprint and for both men's and women's 200 m and 400 m events using the same methodology (Eqs. 3–6 and Fig. 3). Equations 5 and 6 were applied to these approximate contributions to parametrize the model and test its consistency. The details of this parametrization for each running distance are presented in supplementary material.

After parameterization, we formalized a general model for men’s and women’s 100 m, 200 m, and 400 m sprints. To ensure parsimony and simplicity, we fixed the parameters ${k}_{1}$, ${k}_{2}$, ${\mu }_{al}$, and ${\upsigma }_{al}$. Thus, variations in ${P}_{la,max}\left(T\right)$ and ${P}_{al,max}\left(T\right)$ alone shape the model’s time course across sprint duration $T$. We set MAP at 24.5 W kg^−1^ for men’s and 21.0 W kg^−1^for women’s. We assigned ${k}_{1}$, ${k}_{2}$, ${\mu }_{al}$, and ${\sigma }_{al}$ as 2.75 s, 35 s, 1, and -0.4, respectively. We selected these parameters from theoretical considerations outlined in the previous sections and to achieve a reasonable balance based on the optimal parametrization of each metabolic contribution of the men’s and women’s 100 m, 200 m, and 400 m performances.

### Applying the formalized sprint bioenergetics model

**Fig. 4:**
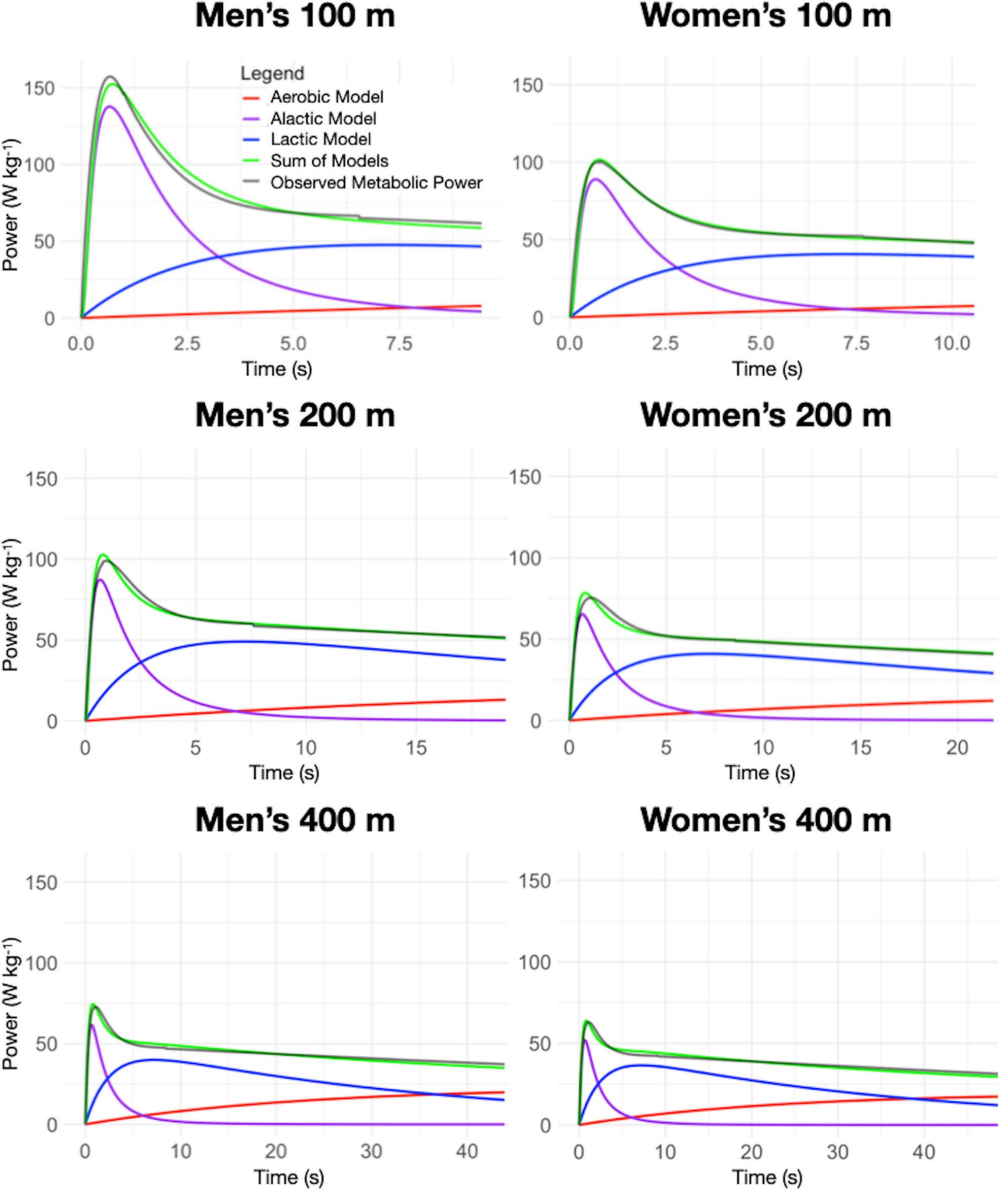
Application of the sprint bioenergetics model to observed metabolic power from di Prampero et al.’s (2005; 2018) methods and Graubner and Nixdorf’s (2011) data. Purple, blue and red lines depict alactic, lactic, and aerobic power, respectively. Green lines denote the combined bioenergetics model, and black lines reflect observed metabolic power. Panels display: Upper-left: Men’s 100 m (${\text{P}}_{\text{al},\text{max}}$: 137.7 W kg^−1^, ${\text{P}}_{\text{la},\text{max}}$: 47.6 W kg^−1^); Upper-right: Women’s 100 m (${\text{P}}_{\text{al},\text{max}}$: 89.0 W kg^−1^, ${\text{P}}_{\text{la},\text{max}}$: 40.7 W kg^−1^); Middle-left: Men’s 200 m (${\text{P}}_{\text{al},\text{max}}$: 87.2 /kg^−1^, ${\text{P}}_{\text{la},\text{max}}$: 49.1 W kg^−1^); Middle-right: Women’s 200 m (${\text{P}}_{\text{al},\text{max}}$: 65.2 W kg^−1^, ${\text{P}}_{\text{la},\text{max}}$: 40.9 W kg^−1^); Lower-left: Men’s 400 m (${\text{P}}_{\text{al},\text{max}}$: 61.5 W kg^−1^, ${\text{P}}_{\text{la},\text{max}}$: 39.9 W kg^−1^); Lower-right: Women’s 400 m (${\text{P}}_{\text{al},\text{max}}$: 52.0 W kg^−1^, ${\text{P}}_{\text{la},\text{max}}$: 36.6 W kg^−1^)

We applied the formalized sprint bioenergetics model to calculate the metabolic power for men’s and women’s 100 m, 200 m, and 400 m events. We performed non-linear least-squares fits to align the model with the observed metabolic power, enabling the determination of ${P}_{la,max}\left(T\right)$ and ${P}_{al,max}\left(T\right)$ for these events. Figure 4 shows the adjusted model compared to the observed data for each sprint performance. Table 2 summarizes fit metrics, including adjusted *R*^2^, total energy, peak power, and distance traveled per sprint, comparing predicted and observed metabolic power, derived from world-record velocity time courses.

**Table 2:** Metrics comparing the sprint bioenergetics model to observed metabolic power from di Prampero et al.’s (2005; 2018) methods and Graubner and Nixdorf’s (2011) data

	Men’s 100 m	Men’s 200 m	Men’s 400 m	Women’s 100 m	Women’s 200 m	Women’s 400 m
Adjusted *R*^2^	0.95	0.97	0.94	0.98	0.96	0.98
Total Energy (J kg^−1^)	766	1171	1925	636	1076	1870
Energy Difference (%)	−0.77	−0.09	−0.88	−0.43	0.05	−1.07
Peak Power (W kg^−1^)	152.28	102.70	74.42	101.65	78.24	63.86
Power Difference (%)	−3.1	3.89	2.34	1.04	3.97	1.38
Distance (m)	98.52	195.10	385.43	96.21	197.25	390.59
Distance Difference (%)	−1.4	−0.3	−1.04	−0.65	−0.03	−1.25

Percentage energy differences reflect model predictions vs. observed power. Distance traveled derives from an algorithm computing distance, velocity, and acceleration from metabolic time series (see text)

The model fits observed data well, with adjusted *R*^2^ values ranging between 0.94 and 0.98, depending on the sprint distance. The total energy expanded estimated by the model aligns with the energy calculated using the metabolic power derived from di Prampero et al.’s (2005; 2018) method using available velocity data (see Fig. 2), with percentage differences ranging from −1.07% to 0.05%.

The maximal metabolic power derived from the bioenergetic model shows variations exceeding 1% (in absolute terms) when compared to values obtained from di Prampero et al.’s (2005; 2018) method. These differences can be partially attributed to the fact that peak metabolic power is an interpolated value. The peaks occurs approximately one second after the start of the sprint, while most of the initial data provided by Graubner and Nixdorf (2011) only spans the 3 to 6-s range. As peak metabolic power relies on early acceleration, limited data introduce uncertainty. We selected these parameters to balance model fit across men’s and women’s sprint performances (see supplementary material for details).

From the metabolic power data, we developed an algorithm to estimate velocity, distance, and acceleration from a metabolic power time series. The algorithm iterates through the power time series to update velocity. It uses prior time steps, acceleration, and power, starting at 0 m s^−1^ and 0 m s^−2^ for velocity and acceleration, respectively. Based on di Prampero et al.’s (2005; 2018) power, the algorithm minimizes the difference between the calculated power (based on velocity and acceleration) and power derived from the sprint bioenergetic model. Applying the algorithm retroactively to power time series derived from velocity data (Fig. 3) results in a distance estimation that is accurate to within 0.01% of the actual distance traveled.

With the algorithm, we estimated the distance covered during each performance from the model’s metabolic power time series. We then compared these estimated distances to those calculated using velocity data from men’s and women’s sprint events reported by Graubner and Nixdorf (2011) (see Fig. 1 caption). The estimated distances, based on the bioenergetic model, are shown in Table 2. Percentage differences between estimated and modeled distances closely match, with variations ranging from 0.03% to 1.4%, depending on the event’s distance. Given assumptions in the modeled velocity time course, using Graubner and Nixdorf’s (2011) data, as well as the limited number of data points, the estimated distances do not perfectly align with the actual distances of 100, 200, and 400 m.

As described in the caption of Fig. 4, ${P}_{la,max}$ exceeds 100 m values in the 200 m sprint: 49.1 W kg^−1^ (men’s 200 m) vs. 47.6 W kg^−1^ (men’s 100 m), and 40.9 W kg^−1^ (women’s 200 m) vs. 40.7 W kg^−1^ (women’s 100 m). For the 400 m event, ${P}_{la,max}$ drops to 39.9 W kg^−1^ (men) and 36.6 W kg^−1^ (women). This trend may be attributed to the increased reliance on ATP and PCr splitting in 100 m, triggering glycolysis inhibition and restricting muscle power production via glycolytic pathways (di Prampero 1981; Girard et al. 2011; Hargreaves & Spriet 2020; Heck et al. 2003; Mader 2003). In the 400 m event, energy demand decreases after the acceleration phase. However, the glycolytic energy contribution remains important enough to cause a progressive decline in power from glycolytic pathways, which may contribute to the gradual reduction in velocity as the race progresses (Heck et al. 2003).

Glycolysis has a maximal theoretical energy production rate (di Prampero 1981; Mader 2003). Studies report this rate at approximately 1 mmol L^−1^ s^−1^ lactate (Heck et al. 2003; Mader 2003), corresponding to a maximal glycolytic power 62 W kg^−1^, about double the maximum aerobic power (di Prampero 1981). The maximal anaerobic lactic powers calculated by the model fits within this experimental range.

Table 3 summarizes energy system contributions for 100, 200 and 400 m sprints. The anaerobic-to-aerobic energy percentage ratios are 95/5 (100 m), 88/12 (200 m), and 70/30 (400 m), and for women’s sprints, 93/7, 86/14, and 69/31. These match Gastin’s (2001) ratios for similar durations: 94/6 (10 s), 82/18 (20 s), and 63/17 (45 s). These ratios should be interpreted with caution, as Gastin’s data exhibits high variability (± 10%) and is derived from a range of methods, including laboratory experiments and mathematical models (Gastin 2001).

**Table 3:** Energy contributions of three energy systems per sprint distance. Percentage values reflect total energy expended per sprint

Metric	Men’s 100 m	Men’s 200 m	Men’s 400 m	Women’s 100 m	Women’s 200 m	Women’s 400 m
Alactic energy (J kg^−1^)	362	239	170	237	179	144
Lactic energy (J kg^−1^)	364	790	1189	357	743	1155
Aerobic energy (J kg^−1^)	40	142	566	42	153	571
Total energy (J kg^−1^)	766	1171	1925	636	1076	1870
% Alactic	47	20	9	37	17	8
% Lactic	48	67	62	56	69	62
% Aerobic	5	12	29	7	14	31

Gastin (2001) estimated alactic contributions at ~ 20–25% of total energy for 25 s sprints and ~ 15% for 50 s sprints. These exceed our model’s estimates of ~ 20% for 20 s sprints and ~ 10% for 50 s sprints (Table 3). Accurate evaluation and measurement of anaerobic alactic energy expenditure experimentally remains however a challenging task subject to high variability (di Prampero 1981).

From Table 3 lactic energy value and prior theoretical considerations, we estimated blood lactate accumulation for each sprint performance (di Prampero and Osgnach 2018). We estimated blood lactate concentrations of 7.5 to 8 mmol L^−1^ for the 100 m, 14.5 to 15 mmol L^−1^ for the 200 m, and 20.5 to 22 mmol L^−1^ for the 400 m, for men and women, respectively. These predictions match experimental blood lactate data (Heck et al. 2003; Hirvonen et al. 1992, 1987; Kindermann 1977). These values also support adopting different blood lactate accumulation anaerobic energy equivalents for men and women. As mentioned previously, female exhibit lower anaerobic lactic energy production, their blood lactate accumulation is similar to the one estimated for men, as expected in theory (Korhonen et al. 2003; Lacour et al. 1990).

Aerobic power also affects energy contribution ratios (Table 3). We modeled the contribution of aerobic metabolism as a rising exponential function, assuming a constant maximal aerobic power (MAP) and a fixed time constant. Yet, aerobic metabolism could contribute more significantly, depending on the athlete, or exhibit faster kinetics based on the sprint distance (di Prampero and Osgnach 2018; Fox et al. 1980; Gastin 2001; Gastin et al. 1995). Nonetheless, despite this limitation, the model offers reasonably accurate and coherent estimations of the contributions of aerobic, anaerobic alactic, and lactic power, when compared to experimental observations and theoretical considerations. Yet, the model currently requires known ${P}_{al,max}$ and ${P}_{la,max}$ values for each sprint.

## Maximal anaerobic lactic and alactic capacities

In the next sections, we modeled ${P}_{al,max}$ and ${P}_{la,max}$ for any sprint duration, based on an individual’s maximal anaerobic alactic and lactic capacities. We based this model on a hypothetical male athlete with sprint times of 9.58 s (100 m), 19.19 s (200 m), and 44.06 s (400 m).

We calculated alactic and lactic contributions for these performances using the sprint bioenergetics model (Fig. 4, Tables 2, 3). These revealed energy trends with sprint duration, guiding functions to model contributions from maximal capacities. Since this hypothetical athlete’s data comprises only three data points, we incorporated theoretical considerations to supplement the limited dataset.

### Maximal alactic capacity and maximal alactic power over time

**Fig. 5:**
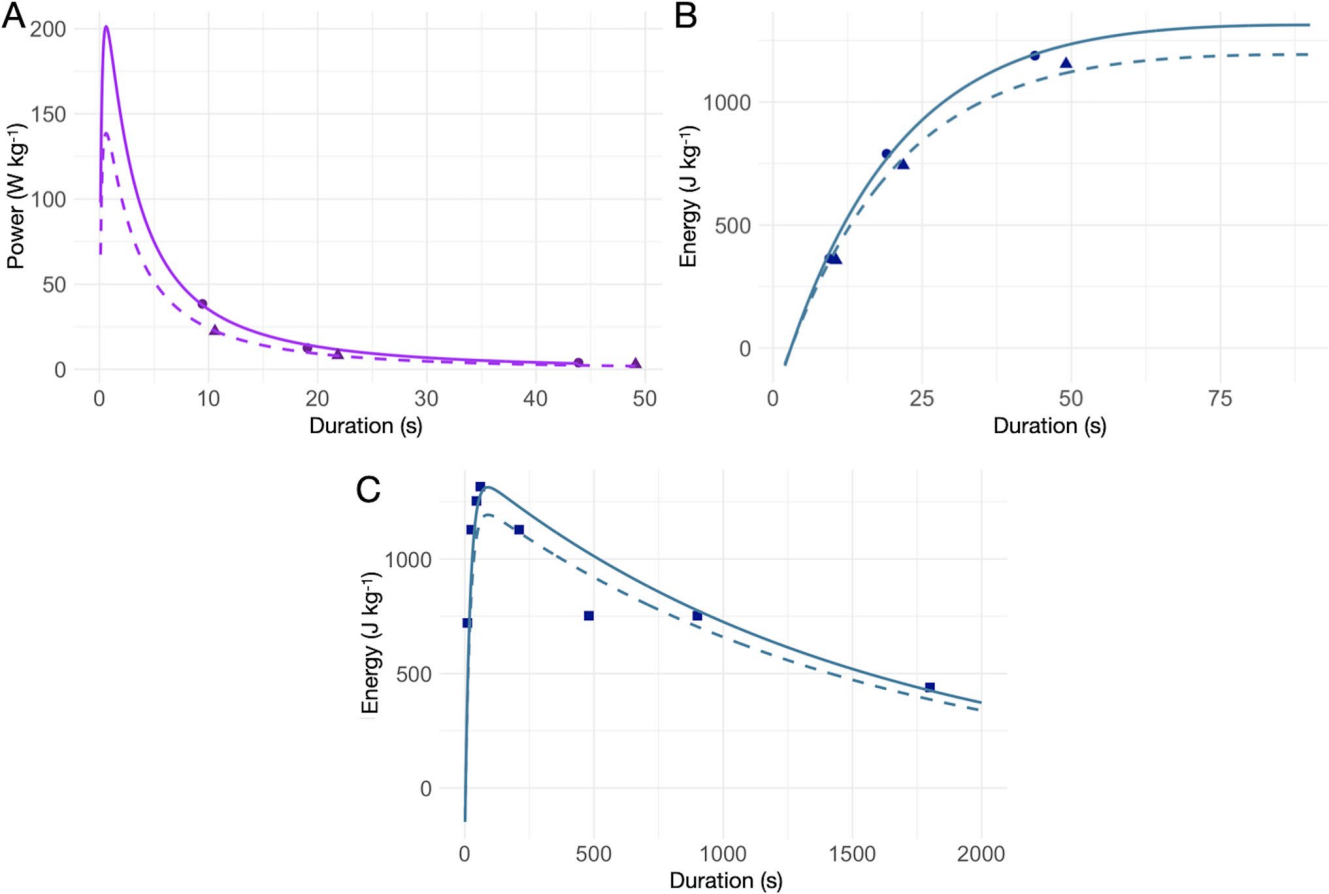
Upper-left panel shows average alactic power vs. sprint duration, with solid points for men’s and triangles for women’s alactic energy derived from the sprint bioenergetics model (100, 200, and 400 m). Solid and dashed lines depict modeled alactic energy, for men and women, respectively. The model estimates maximal alactic capacities at 376 J kg^−1^ (men) and 259 J kg^−1^ (women). Upper-right panel displays lactic energy vs. sprint duration, with solid points and triangles for men’s and women’s data from the sprint bioenergetics model. The solid (men) for men and dashed (women) lines show the modeled lactic energy as a function of time, with estimated maximal lactic of 1314 J kg^−1^ (men) and 1194 J kg^−1^ (women). Lower panel extends these lactic energy estimates for longer running duration, with solid and dashed lines representing men’s and women’s model respectively. Squares denote lactic energy Kindermann’s (1977) blood lactate data

Maximal energy rate from PCr and ATP splitting peaks at muscle contraction onset, declining around exercise durations of 1 s (Gastin 2001). It is also suggested that the highest energy contribution from ATP and PCr splitting takes place during running exercises lasting approximately 6 s (di Prampero 1981; Gastin 2001). Therefore, the contribution of alactic energy as a function of race duration cannot be explained by an exponential decay function, as it would predict anaerobic alactic energy exceeding the maximum available for exercise duration lasting less than 3 s. Figure 5 shows the trend of average alactic power over sprint duration. We modeled the trend of average alactic power over sprint duration, ${P}_{al, avg}\left(T\right)$, with the following log–normal function.

8 $${P}_{al, avg}\left(T\right)=\frac{{E}_{al,max}}{T}{\bullet e}^{\left(\frac{{-\left(\text{ln}\left(T\right)- 1.75\right)}^{2}}{2{\left(1.5\right)}^{2}}\right)}$$

where ${E}_{al,max}$ is the maximal alactic capacity in J kg^−1^. Given limited data, we set the parameters $\mu$ and $\sigma$ of the log–normal function to 1.75 and 1.5, to minimize the standard residual error (1.386 W kg^−1^) with the men’s average alactic energy expenditure over the 100, 200 and 400 m, while remaining coherent to the theoretical considerations stated previously. With the set parameters, the model aligns with theory, with a maximal anaerobic alactic power attained around 0.6 s. The maximal alactic power decreases significantly for exercises lasting more than 1 s (Gastin 2001). Maximal anaerobic energy expenditure is observed for exercise duration of 4 to 6 s (di Prampero 1981). We estimated a maximal anaerobic alactic capacity of 376 J kg^−1^ from non-linear least-squares fitting of Eq. 8 to the available data for the 100 m, 200 m, and 400 m events. The calculated maximal alactic capacity falls within the theoretical range of maximal alactic capacity (310 to 390 J kg^−1^) (di Prampero and Osgnach 2018). Because of limited data, more sprint observations are needed to validate Eq. 8 and refine its parameters.

Equation 8 models alactic power and energy contributions over total sprint duration. Using energy expended in the sprint, we calculated ${P}_{al,max}\left(T\right)$ to match the integral of the alactic power model (Eq. 6) with expected alactic energy over the duration ($T$). This problem can be solved numerically.

### Maximal lactic capacity and maximal lactic energy over time

In maximal effort sprints of different durations, lactate accumulation lactate rises with sprint duration until it reaches a peak, corresponding to running distances completed within the duration of 50 to 100 s (di Prampero and Osgnach 2018; Kindermann 1977; Lacour et al. 1990). Over such durations, blood lactate concentration reaches approximately 20–25 mmol L^−1^ (Heck et al. 2003; Kindermann 1977; Lacour et al. 1990), reflecting 1250 to 1500 J kg^−1^ of lactic energy expenditure. For longer running exercises, lasting 1 h, nearly half of this maximal anaerobic lactic energy is used (2011).

To approximate this behaviour, we modeled total lactic energy expenditure with a bi-exponential function, Eq. 9. Given limited data, we set the bi-exponential time constants to 20 s (rise) and 1500 s (decay). We chose these parameters to: 1) minimize standard error of anaerobic lactic energy expenditure in the men’s 100 m, 200 m, and 400 m events, as calculated using the bioenergetic model of sprint running, 2) ensure that the maximum value of Eq. 9 occurs between 50 and 100 s, and 3) align the model with the trend of estimated lactic energy expenditure, based on blood lactate concentrations across various running distances up to 10,000 m, as reported by Kindermann (1977). Also, we assumed that for exercises lasting less than 3 s, the energy contribution from anaerobic lactic metabolism is negligible (di Prampero 1981; Saltin and Essén, 1971), per Eq. 9’s time adjustment. This assumption relies on the hypothesis that, within such a short time frame, the rate of PCr and ATP breakdown reaches its peak, rapidly leading to a reduced rate of ATP resynthesis or utilization by the muscle (Gastin 2001; Hermansen 1981). Consequently, this is thought to result in a faster glycolysis inhibition than its activation kinetics. The parameterization of variables in Eq. 9 remains exploratory and could be refined further with additional data on lactic energy expenditure across different running distances, from 100 m to marathon events.

9 $${E}_{la}(T)={E}_{la,max}\bullet {k}_{norm,2}\bullet \left({1-e}^{\frac{-\left(T - 3\right)}{20}}\right)\bullet {e}^{\frac{-\left(T-3\right)}{1500}}\text{for T}> 3\text{ s}$$

${k}_{norm,2}$ is a normalization constant, as before, based on the time constants (20 and 1500 s) of the bi-exponential function of Eq. 9. We calculated a maximal lactic capacity of 1314 J kg^−1^ by applying a non-linear least squares fit of Eq. 9 to the calculated anaerobic lactic energy (${E}_{la}$) over the 100, 200 and 400 m sprints. The maximal lactic capacity was achieved at approximately 90 s. For a male athlete, this energy corresponds to a lactate accumulation of 20.9 mmol L^−1^, a reasonable value, consistent with experimental lactate accumulation measurements in elite athletes (Heck et al. 2003; Kindermann 1977). Figure 5 shows the bi-exponential fit of the anaerobic lactic energy contribution for 100 m, 200 m, and 400 m races, as calculated by the model. Figure 5 extends this model to longer durations, comparing it against Kindermann’s (1977) blood lactate data. As before, we assumed that, for male athlete, a blood lactate accumulation of 1 mmol L^−1^ is equivalent to the consumption of 3 mL O₂ kg^−1^ of body mass (di Prampero 1981; di Prampero and Ferretti 1999).

Using lactic energy estimates from Eq. 9, we calculated blood lactate levels for longer durations. We estimated blood lactate concentrations of approximately 14, 8, and 1.7 mmol L^−1^ for running duration of 900 and 1,800 and 7200 s, respectively. These values align closely with blood lactate values reported in the literature of approximately 13, 8, and 2.5 mmol L^−1^ for similar running duration (Kindermann 1977). Figure 5, Kindermann’s (1977) data deviate from our lactic energy model over durations around 200 and 500 s. This discrepancy may partly result from the assumption that 1 mmol L^−1^ of lactate corresponds to the consumption of 3 mL of O₂ per kilogram of body mass (Graubner and Nixdorf 2011). As discussed in the section on anaerobic lactic energy contribution, lactate space, influenced by body composition, potentially affects the energy equivalence of lactate accumulation (Taylor and Péronnet 1981). Athletes specialized in various events have different body and muscle fibre compositions (Stachoń et al. 2023), suggesting that the energy equivalent of lactate accumulation may vary with race duration. While the model does not fully match experimental data, quantifying lactate accumulation and anaerobic lactic energy contributions remains an approximate method to quantify glycolytic energy contribution and varies across studies (di Prampero 1981; Gastin 2001). Equation 9 lays groundwork for future research on lactic energy dynamics in longer running distances.

As before, we calculated lactic energy contributions over total sprint duration using the model. We determined ${P}_{\text{la,max}}\left(T\right)$ to match Eq. 5’s integral with expected lactic energy over $T$. This problem can be solved numerically.

## Application of the model

Although relationships from the section on maximal capacities allow an estimation of lactic and alactic energy contributions based on race duration and maximal lactic and alactic capacities, they remain exploratory and are constrained by limited data. Yet we tested their application alongside the bioenergetic model of sprint running, describing the time course of energy dynamics for the three metabolic systems during sprints We evaluated this comprehensive model’s (formulated in supplementary material) ability to extract maximal lactic and alactic energy capacities from running performances and to predict the energy contributions of alactic, lactic, and aerobic metabolisms across various sprint distances. To achieve this, we considered two hypothetical athletes: (i) a male athlete with performances matching the men’s results reported by Graubner and Nixdorf (2011) for the 100 m (9.58 s), 200 m (19.19 s), and 400 m (44.06 s); (ii) a female athlete with performances equivalent to the women’s results reported by Graubner and Nixdorf (2011) for the 100 m (10.73 s), 200 m (22.02 s), and 400 m (49.32 s). We parametrized Eqs. 8 and 9 using alactic and lactic energy data from men’s performances. We assumed identical parameters ($\mu$ and $\sigma$ for Eq. 8, and rise/decay time constants for Eq. 9) for both male and female hypothetical athletes.

Dashed lines in Fig. 5 show the alactic and glycolytic fits derived from anaerobic alactic average power and lactic energy calculations based on the women’s performances. With this approach, the only factors distinguishing the two hypothetical athletes (male and female) are their maximal aerobic, anaerobic alactic and anaerobic lactic capacities. We optimized these to align with each athlete’s best performances across sprint distances. We implemented an optimization algorithm to find alactic and lactic capacities minimizing distance error for each sprint duration. We included reaction times from Graubner and Nixdorf (2011) in each performance calculation. For this optimization, we reused the algorithm calculating distance, velocity, and acceleration from the metabolic power time series. We set the maximal aerobic power (MAP) at 24.5 W kg^−1^ for the male athlete and 21 W kg^−1^ for the female athlete.

Table 4 presents optimization results. The maximal alactic capacity was 328 J kg^−1^ for the male athlete and 218 J kg^−1^ for the female athlete, and the maximal lactic capacity was 1460 J kg^−1^ for the male and 1295 J kg^−1^ for the female. These values fall within the expected ranges (310–390 J kg⁻^1^) for males. For females, the value falls slightly outside the theoretical estimated range (230–292 J kg⁻^1^). The optimization also suggests a lactate accumulation of 23 mol L^−1^ during an 800 m race for both athletes, consistent with data on elite athletes (Heck et al. 2003; Lacour et al. 1990). The male’s combined anaerobic capacity of 1787 J kg⁻^1^ aligns with Péronnet and Thibault’s (1989) analysis of 1987 World Records, individual performances, and projections, estimating anaerobic capacities ranging between 1650–1750 J kg^−1^ for men and between 1500–1660 J kg^−1^ for women.

**Table 4:** Distance traveled and energy contribution of the 3 metabolisms over 100, 200, and 400 m for hypothetical male and female athletes, based on a male athlete with a maximal aerobic capacity of 24.5 W kg^−1^, a maximal alactic capacity of 328 J kg^−1^ and lactic capacity of 1460 J kg^−1^, and a female athlete with a maximal aerobic capacity of 21 W kg^−1^, a maximal alactic capacity of 218 J kg^−1^ and a maximal lactic capacity of 1295 J kg^−1^

	Men’s 100 m	Men’s 200 m	Men’s 400 m	Women’s 100 m	Women’s 200 m	Women’s 400 m
Distance (m)	99.99	201.87	399.99	99.99	205.39	391.21
Error (%)	− 0.00	0.93	− 0.00	− 0.00	2.69	− 2.20
Total (J kg^−1^)	779	1237	2027	679	1139	1864
Lactic (J kg^−1^)	429	856	1327	436	838	1214
Alactic (J kg^−1^)	311	238	131	201	147	79
Aerobic (J kg^−1^)	40	142	566	42	154	572

As discussed previously, the bioenergetic model of sprint running, which provides the time course of energy contributions from each metabolic pathway during a race, underestimated the distance traveled. This underestimation can be attributed to the limited number of observations available in the study by Graubner and Nixdorf (2011) and the assumptions made to model the velocity time course using this data. The optimization procedure adjusts this underestimation by favouring higher anaerobic lactic capacities than those calculated when analyzing each distance individually. This adjustment stems from a greater underestimation of distance in the 400 m event compared to shorter sprints of 100 m and 200 m (refer to Fig. 1 caption).

Despite its limitations, the optimization approach minimizes the distance error across the 100, 200, and 400 m sprints. It achieves a mean absolute distance error of 0.31% for the hypothetical male athlete and 1.63% for the female athlete. The larger percentage error for the female stems from the parametrization of Eqs. 8 and 9, which was conducted using men’s calculated anaerobic alactic and lactic energy and then directly applied to women. Indeed, the residual standard error for the fit of lactic energy with respect to race duration (Eq. 9) was approximately twice as high for women’s data compared to men’s (45.6 vs. 21.3 W kg^−1^). Consequently, lactic energy was underestimated for women’s 400 m performances, potentially contributing to the observed discrepancy in distance estimation. Despite differences in anaerobic lactic energy contributions previously discussed, the energy contributions across distances presented in Table 4 are consistent with individual race performance analysis (refer to Table 3).

We simulated the performances of hypothetical male and female athletes over 60, 100, 200, 300, and 400 m, based on their maximal anaerobic lactic and alactic capacities. Assuming a reaction time of 0.15 s, we used an optimization algorithm to minimize running time for each distance. Table 5 lists simulated performances of these athletes.

**Table 5:** Simulated sprint times for the hypothetical male and female athletes

Distances (m)	Men’s simulated times (s)	Women’s simulated times (s)
60	6.29	7.04
100	9.58	10.73
200	19.01	21.39
300	30.44	34.66
400	44.05	50.85

The simulation is based on a male athlete with a maximal aerobic capacity of 24.5 W kg^−1^, a maximal alactic capacity of 328 J kg^−1^ and lactic capacity of 1460 J kg^−1^, and a female athlete with a maximal aerobic capacity of 21 W kg^−1^, a maximal alactic capacity of 218 J kg^−1^ and a maximal lactic capacity of 1295 J kg^−1^

Usain Bolt has no official 60 m time but ran 300 m in 30.97 s in 2010. Using our model, we estimated the anaerobic capacities of a hypothetical athlete matching Bolt’s 100 m and 200 m performances. The model suggests Bolt could have run 60 m in 6.29 s, faster than the World Record of 6.34 s. Official data from the 2009 World Championships indicates Bolt’s 60 m split in his 100 m World Record was 6.31 s (Graubner and Nixdorf 2011).

From the simulation, we estimated a time of 30.44 s for the men’s 300 m, 0.8% faster than the 30.69 s World Record. However, the 300 m is not a standard event in major championships. that whereas ~ 50% of sprinters compete in both the 100 m and 200 m, only ~ 20% do both the 200 m and 400 m (Brustio et al. 2023). This reflects distinct physiological demands across these sprints. The 100 m and 200 m rely heavily on anaerobic energy production. The alactic energy system accounts for ~ 40% of the total energy contribution in the 100 m sprint and ~ 20% in the 200 m sprint. Meanwhile, the lactic energy system contributes about ~ 55% of the total energy for the 100 m and ~ 70% for the 200 m. In the 400 m sprint, in absolute terms, more lactic energy is spent than in shorter sprint (see Table 4), but the proportion of lactic energy contribution remains similar to that of shorter sprints, ~ 60%, whereas the alactic energy contribution drops to roughly 8%, indicating that longer sprint events rely less on alactic energy production. The hypothetical athlete, modeled on world-class performances across all three sprints, shows optimized capacity to deliver both alactic and lactic energy compared to actual athletes competing in the 300 m, which could explain the fast 300 m performance estimate.

For women, the analysis provides similar insights. Despite performances at the 2009 World Championships falling short of World Records for the 100 m (10.49 s), 200 m (21.34 s), and 400 m (47.60 s), the estimated 60 m time of 7.04 s is only 0.12 s slower than the 6.92 s World Record by Irina Privalova. Privalova’s 100 m best of 10.77 s is comparable to Shelly-Ann Fraser-Pryce’s 10.73 s in 2009. The estimated 300 m time for women is 34.66 s is close to the 34.41 s world record. Yet this can be explained as the 300 m is not a standard distance run in major championships. The 400 m estimated time is over a second slower than Williams time at the 2009 World Championships This likely stems from underestimating lactic in women’s simulation, tied to Eq. 9’s parametrization.

## Discussion and critique of the methods

We developed a sprint bioenergetics model to estimate maximal anaerobic alactic and lactic capacities, as well as the simulations used to predict optimal performances over sprint distances, are based on assumptions that have been analyzed and contextualized with the scientific literature in the previous section. The following section revisits and further discusses some of these assumptions to highlight the limits and key areas for future work.

### Metabolic power estimations

The methods proposed by di Prampero et al. (2005; 2018) rely on key assumptions and simplifications. For a detailed critique and discussion of this approach, readers are referred to the original publications (di Prampero et al. 2015, 2005, 2023; di Prampero and Osgnach 2018; Osgnach et al. 2023). Briefly, the method is based on the assumption that (i) the overall mass of the runner is condensed in his/her centre of mass. This necessarily implies that (ii) the energy expenditure due to internal work performance (for moving the upper and lower limbs with respect to the centre of mass) and hence that (iii) the stride frequency is the same during both accelerated running and uphill running at constant velocity up the appropriate “Equivalent Slope". External factors such as wind, track curvature, and the influence of other runners on the track are not accounted for. The energy required to overcome air resistance in windless conditions is calculated as 0.01 times the square of the runner’s instantaneous velocity (m s^−1^), where the constant 0.01 is a coefficient determined by Pugh (1970) and di Prampero (1986).

Additional assumptions involve adjustments to Minetti (2002) addressing extreme slopes and energy costs observed during the acceleration phase of male elite sprinters (di Prampero and Osgnach 2018). Another simplification is the assumption that, following the acceleration phase, the energy cost of running stabilizes at a constant value corresponding to the cost of running on flat terrain.

The adjustments to Minetti et al. (2002) could impact the estimation of peak metabolic power and explain the observed difference (> 1% in absolute value) between the peak metabolic powers derived from the bioenergetic model and the ones derived from the energy cost-based approach using Minetti et al.’s (2002) equation. To further investigate this, we compared the original approach based on Minetti et al.’s (2002) equation—adjusted by di Prampero and Osgnach (2018)—with the more recent formulation proposed by Minetti and Pavei (2018), which extends the validity range of the cost function up to forward accelerations of 8.24 m·s⁻^2^ using data from Giovanelli et al. (2016). The following equation proposed by Minetti and Pavei (2018) provides an expression to compute the cost of accelerated running (${C}_{AR}$, in J kg^−1^) from forward acceleration ($a$, in m s^−1^).

10 $${C}_{AR}=0.102\bullet {\left({a}^{2}+96.2\right)}^{0.5}\bullet (4.03 a+3.6\bullet {e}^{-0.408 a})$$

Substituting the updated equation into our computations resulted in lower peak metabolic power estimates, particularly for the 100 m events, with differences reaching 11% in men and 5% in women compared to the original method. Interestingly, for the 200 m and 400 m events, the differences between the two approaches remained under 1%. When comparing the bioenergetic model-derived peak metabolic powers to those obtained using the updated approach, a slightly smaller absolute difference is observed in the men’s 100 m race (2.52% vs. 3.1%) compared to the previously reported difference using the original method. In contrast, for the women’s 100 m race, a larger difference is observed (3.56% vs. 1.04%). For both the 200 m and 400 m events, similar percentage differences persist between the peak metabolic powers derived from the bioenergetic model and those calculated using the two velocity and acceleration-based approaches.

These findings suggest that the observed discrepancy (> 1% in absolute terms) between metabolic power values derived from the bioenergetic model and indirect power estimations using energy cost-based approaches, may primarily stem from limitations in the velocity data used to estimate peak metabolic power, which occurs within the first second of running. Specifically, the low resolution of the data from Graubner and Nixdorf (2011) likely introduces inaccuracies by requiring interpolation to estimate metabolic peak powers, thereby contributing to the differences observed between the bioenergetic model's peak metabolic power output and that derived from velocity and acceleration data, whether using Minetti et al.'s (2002) equation or Minetti and Pavei's (2018) equation. Future research should focus on methods using higher-resolution velocity and acceleration measurements to enhance the accuracy of metabolic power and distance-covered estimations (see Fig. 1). Recalibrating the bioenergetic model using these advanced measurements could significantly reduce discrepancies between metabolic power derived from the bioenergetic model and that calculated through energy cost-based approaches, such as those proposed by di Prampero and Osgnach (2018) or Minetti and Pavei (2018). Additionally, incorporating advanced technologies, such as force plates combined with image analysis, could address these limitations, enhance the precision and help further refine this indirect approach relying on energy cost (di Prampero et al. 2005).

### Interpretation of alactic energy expenditure

The sprint bioenergetics model depends on several assumptions to estimate the anaerobic alactic and lactic energy expended during sprints. Regarding anaerobic alactic energy, the assumptions, leading to an estimated maximal anaerobic alactic capacity (310–390 J kg^−1^ for males, 230–292 J kg^−1^ for females), are based on the percentage of active muscle mass relative to total body weight (20–25% for males and 15–20% for females). These values are approximations and subject to interindividual variability (Janssen et al. 2000; Miller et al. 2024). The quantity of high-energy phosphates can also be significantly influenced by an individual’s capacity to recruit muscle fibres and their specific characteristics, which vary from one person to another (di Prampero 1981; Hirvonen et al. 1992). Moreover, we assumed that the level of PCr is the same at the beginning of every sprint. However, studies indicate that warm-up exercises may lower muscle PCr concentrations before the start of a sprint (Hirvonen et al. 1987). Pre-race activities could therefore affect anaerobic alactic energy availability.

### Interpretation of lactic energy expenditure

We assessed lactic energy expenditure by comparing blood lactate accumulation to the anaerobic lactic energy equivalent: 3 mL O_2_ kg^−1^ of body mass for accumulated blood lactate of 1 mmol L^−1^ in men, and 2.7 mL O_2_ kg^−1^ of body mass in women. This method offers an approximation to evaluate energy expenditure through glycolytic pathways. The assumptions underlying these energy equivalents are thoroughly discussed by di Prampero (1981). Several factors can influence the estimation of lactic energy through lactate accumulation. For instance, lactate produced in muscles can be metabolized during the initial minutes of recovery, prior to the measurement of blood lactate accumulation (di Prampero 1981). Additionally, the distribution of lactate in body fluids can vary depending on tissues, the muscle groups involved, and individual water distribution in the body (di Prampero 1981). Therefore, factors such as an athlete’s muscle fibre composition and body composition, which impact the available lactate space and consequently the energy equivalent, can influence the interpretation of glycolytic energy derived from lactate accumulation (di Prampero 1981; Taylor and Péronnet 1981).

Given these considerations, the approximation of an anaerobic lactic energy equivalent—3 mL O_2_ kg^−1^ of body mass for men and 2.7 mL O_2_ kg^−1^ of body mass for women—for accumulated blood lactate of 1 mmol L^−1^ remains a reasonable method (di Prampero 1981). We used this relation to validate whether lactic energy expenditure leads to realistic accumulated blood lactate based on existing experimental data.

### Modelling approach

To develop our sprint bioenergetics model, we assumed, for simplicity, that the anaerobic alactic power contribution follows a log–normal function, the lactic power follows a bi-exponential function, and the aerobic power contribution follows an exponentially rising function. We also assumed these three functions operate independently, summing to total power production. However, this approach does not fully reflect physiological reality, as evidence shows metabolic components interact through complex dynamic processes (di Prampero 1981; Heck et al. 2003; Mader 2003).

A more accurate representation could be achieved by adopting a dynamic model incorporating differential equations to capture the feedback and interactions between the metabolic systems. Mader (2003) proposed such a model, emphasizing the role of ADP concentration, resulting from the utilization of high-energy phosphates, in regulating the glycolytic and aerobic metabolism contributions.

Future research should focus on adapting and applying this dynamic model to metabolic power derived from sprinting data. Doing so would provide a more comprehensive understanding of the interactions between energy systems within our sprint bioenergetics model.

### Energy expenditure and maximal capacities

Our analysis presents a preliminary approach to estimate maximal anaerobic alactic and lactic capacities, using theoretical considerations alongside available data from the 100, 200, and 400 m sprints. A key observation from this analysis is that, regardless of whether the athlete is competing in the 100, 200, or 400 m events, energy expenditure from the anaerobic alactic and anaerobic lactic systems always never reaches the estimated maximal capacity. Equation 9 indicates maximal lactic capacities are fully utilized only in performances lasting ~ 90 s. Given the performances studied are from World Championships, it is reasonable to assume they represent maximal efforts. Morton’s (2006) review of bioenergetic models notes that such models generally apply to activities of maximal intensity or those that ultimately lead to exhaustion. Consequently, a common approach in whole-body bioenergetics modeling is to assume the existence of an energy reserve that represents the athlete’s maximal capacity (Behncke 1993, 1997; Boillet et al. 2024; Moritani et al. 1981; Morton 2006; Skiba et al. 2012, 2015; Weigend et al. 2021). When this energy reserve is fully depleted, the model assumes the athlete has reached exhaustion. The observation that maximal efforts in the 100, 200, and 400 m sprints, as well as maximal efforts beyond 90 s can occur without completely using maximal anaerobic alactic and lactic capacities contradicts this modeling approach and suggests that additional mechanisms need to be considered to accurately model and represent performance. Equations 8 and 9 offer exploratory functions to model energy availability over sprint duration, using the athlete’s maximal capacity. These functions remain basic yet preliminary but provide a starting point for further exploration. Using comparable data for more sprint distances, Eqs. 8 and 9 can be refined to better understand why maximal capacities remain underutilized in maximal sprint efforts.

### Data availability

The current model was developed using only three sprint performances, with the 100 m sprint providing the most precise measurement details (di Prampero and Osgnach 2018). Future research should focus on testing the model’s generalizability across diverse scenarios, including non-elite runners and both shorter (60 m) and longer distances (800 m and 1500 m), to enhance its robustness. At present, the model’s functions and parameters are derived partly from theoretical principles and partly from adjustments aimed at optimizing the goodness of fit with the available data.

Data for longer running distances, such as 800 m and 1500 m, with the level of detail required to apply di Prampero et al.’s (2005; 2018) method to estimate the time course of metabolic power, remain unavailable (di Prampero and Osgnach 2018). Future efforts should prioritize the collection and accessibility of comprehensive datasets with high sampling rates for time splits and velocity measurements. This would enable more refined analyses and foster a deeper understanding of the bioenergetics of running, both through the framework utilized in this article—drawing inspiration from the methods proposed by di Prampero et al. (2005; 2018)—and through new, unexplored approaches.

## Conclusion

The short duration and the significant role played by acceleration makes the analysis of energy contribution from the aerobic, anaerobic lactic and anaerobic alactic metabolisms particularly challenging in sprint events. The nature of sprints prevents the attainment of steady states required for the direct measurements of the respective metabolic contributions. Based on an indirect approach, to estimate metabolic power from the time course of velocity in sprint performance, we have developed a detailed bioenergetic sprint running model, which is comprehensively formulated in supplementary material. Despite its limits and assumptions, the model enables: (1) estimations of maximal lactic and alactic capacities from a set of optimal sprint performances, (2) calculations of aerobic and anaerobic total energy contributions during sprints, and (3) modeling of the time course of power contributions from the different metabolic pathways during a sprint. The model differs from previous ones by simultaneously incorporating the following elements: (1) it separates anaerobic energy contributions into anaerobic alactic and anaerobic lactic sources; (2) it represents the fact that, despite being maximal efforts, performances in the 100 m, 200 m, and 400 m events utilize only a fraction of the available maximal anaerobic alactic and anaerobic lactic capacities. As a final note, these findings underline the value of World Record performances to pursue the knowledge advancement of the bioenergetics of running.

## Supplementary Information

Below is the link to the electronic supplementary material.

Supplementary file1 (DOCX 35 KB)

## Data Availability

The datasets and source code generated and used in the current study have been compiled into an R package named *runrgetics*, which is available in the following public repository: https://github.com/JrmyBriand/runrgetics

## List of symbols

$a\left(t\right)$: Forward running acceleration as a function of time (m s^−^^2^)
ADP: Adenosine diphosphate
AMP: Adenosine monophosphate
ATP: Adenosine triphosphate
BMR: Basal metabolic energy rate (W kg^−^^1^)
${C}_{AR}$: Cost of accelerated running (J kg^−^^1^)
${E}_{al,max}$: Maximal alactic capacity (J kg^−^^1^)
${E}_{la}(T)$: Lactic energy spent as a function of race duration T (J kg^−^^1^)
${E}_{la,max}$: Maximal lactic capacity (J kg^−^^1^)
MAP: Maximal aerobic power per unit body mass (W kg^−^^1^)
${P}_{aer}\left(\text{t}\right)$: Aerobic power contribution per unit body mass as a function of time (W kg^−^^1^)
${P}_{al}\left(t\right)$: Alactic power contribution per unit body mass as a function of time (W kg^−^^1^)
${P}_{al, avg}\left(T\right)$: Average alactic power, per unit body mass, over race duration (T) (W kg^−^^1^)
${P}_{al,max}\left(T\right)$: Peak alactic power over race duration (T)
${P}_{an,accel}(t)$: Lactic power of the acceleration phase per unit body mass (W kg^−^^1^)
PCr: Phosphocreatine
${P}_{la}(t)$: Lactic power contribution per unit body mass as a function of time (W kg^−^^1^)
${P}_{la,max}\left(T\right)$: Maximum lactic power per unit body mass over a race duration T (W kg^−^^1^)
${P}_{tot}\left(t\right)$: Total power per unit body mass (sum of ${P}_{aer}\left(\text{t}\right)$, ${P}_{al}\left(t\right)$ and ${P}_{la}(t)$) (W kg^−^^1^)
${k}_{aer}$: Half-life time constant of the aerobic metabolism, 23 s
${k}_{an}$: Half-life time constant of the anaerobic system during the acceleration phase, 2 s
${k}_{norm}$: Normalization constant of lactic power contribution
${k}_{norm,2}$: Normalization constant of lactic energy over race duration
${k}_{1}$: Time constant of the exponential rise of lactic power contribution (s)
${k}_{2}$: Time constant of the exponential decay of lactic power contribution (s)
$$t$$: Time (s)
$T$: Total sprint duration (s)
v(t): Running velocity as a function of time (m s^–1^)
${v}_{f}$: Peak velocity attained during the race (m s^−^^1^)
VO_2_max: Maximal oxygen consumption (mL O_2_ kg^−^^1^ min^−^^1^)
R^2^: Coefficient of determination
${\mu }_{al}$: Anaerobic alactic constant dictating how fast peak power is achieved
${\sigma }_{al}$: Anaerobic alactic constant dictating how fast peak power decays
$\tau$: Time constant of the velocity exponential rise during the acceleration phase (m s^−^^1^)

## References

[CR1] Alvarez-Ramirez J (2002) An improved Peronnet-Thibault mathematical model of human running performance. Eur J Appl Physiol 86(6):517–525. 10.1007/s00421-001-0555-3 11944100 10.1007/s00421-001-0555-3

[CR2] Arcelli E, Cavaggioni L, Alberti G (2014) Il lattato ematico nelle corse dai 100 ai 1500 metri : confronto fra uomo e donna. Sci & Sport 14(21):48–53

[CR3] Behncke H (1993) A mathematical model for the force and energetics in competitive running. J Math Biol 31(8):853–878. 10.1007/BF00168050 8263429 10.1007/BF00168050

[CR4] Behncke H (1997) Optimization models for the force and energy in competitive running. J Math Biol 35(4):375–390. 10.1007/s002850050057 9104011 10.1007/s002850050057

[CR5] Boillet A, Messonnier LA, Cohen C (2024) Individualized physiology-based digital twin model for sports performance prediction: a reinterpretation of the Margaria-Morton model. Sci Rep 14(1):5470. 10.1038/s41598-024-56042-0 38443504 10.1038/s41598-024-56042-0 PMC10915161

[CR6] Brustio PR, Rainoldi A, Boccia G (2023) Two is better than one: Successful world-class sprinters compete in two disciplines. J Funct Morphol Kinesiol 8(2):52. 10.3390/jfmk8020052 37218847 10.3390/jfmk8020052 PMC10204379

[CR7] Capelli C, Cautero M, di Prampero PE (2001) New perspectives in breath-by-breath determination of alveolar gas exchange in humans. Pflugers Arch 441(4):566–577. 10.1007/s004240000429 11212222 10.1007/s004240000429

[CR8] Cautero M, Beltrami AP, di Prampero PE, Capelli C (2002) Breath-by-breath alveolar oxygen transfer at the onset of step exercise in humans: methodological implications. Eur J Appl Physiol 88(3):203–213. 10.1007/s00421-002-0671-8 12458363 10.1007/s00421-002-0671-8

[CR9] Cavagna GA, Komarek L, Mazzoleni S (1971) The mechanics of sprint running. J Physiol 217(3):709–721. 10.1113/jphysiol.1971.sp009595 5098087 10.1113/jphysiol.1971.sp009595 PMC1331572

[CR10] di Prampero PE (1981) Energetics of muscular exercise. Reviews of physiology biochemistry and pharmacology. Springer, Berlin Heidelberg, pp 143–222 10.1007/BFb0035266 7015457

[CR11] di Prampero PE (1986) The energy cost of human locomotion on land and in water. Int J Sports Med 7(2):55–72. 10.1055/s-2008-1025736 3519480 10.1055/s-2008-1025736

[CR12] di Prampero PE, Ferretti G (1999) The energetics of anaerobic muscle metabolism: a reappraisal of older and recent concepts. Respir Physiol 118(2–3):103–115. 10.1016/s0034-5687(99)00083-3 10647856 10.1016/s0034-5687(99)00083-3

[CR13] di Prampero PE, Osgnach C (2018) The energy cost of sprint running and the energy balance of current world records from 100 to 5000 m. Biomechanics of training and testing. Springer International Publishing, Cham, pp 269–297

[CR14] di Prampero PE, Piiper J (2003) Effects of shortening velocity and of oxygen consumption on efficiency of contraction in dog gastrocnemius. Eur J Appl Physiol 90(3–4):270–274. 10.1007/s00421-003-0947-7 14523564 10.1007/s00421-003-0947-7

[CR15] di Prampero PE, Capelli C, Pagliaro P, Antonutto G, Girardis M, Zamparo P, Soule RG (1993) Energetics of best performances in middle-distance running. J Appl Physiol 74(5):2318–2324. 10.1152/jappl.1993.74.5.2318 8335562 10.1152/jappl.1993.74.5.2318

[CR16] di Prampero PE, Fusi S, Sepulcri L, Morin JB, Belli A, Antonutto G (2005) Sprint running: a new energetic approach. J Exp Biol 208(Pt 14):2809–2816. 10.1242/jeb.01700 16000549 10.1242/jeb.01700

[CR17] di Prampero PE, Botter A, Osgnach C (2015) The energy cost of sprint running and the role of metabolic power in setting top performances. Eur J Appl Physiol 115(3):451–469. 10.1007/s00421-014-3086-4 25549786 10.1007/s00421-014-3086-4

[CR18] di Prampero PE, Osgnach C, Morin J-B, Zamparo P, Pavei G (2023) Mechanical and metabolic power in accelerated running-PART I: the 100-m dash. Eur J Appl Physiol. 10.1007/s00421-023-05236-x 37300700 10.1007/s00421-023-05236-x

[CR19] Elzhov TV, Mullen KM, Spiess A-N, Bolker B (2023) minpack.lm: R Interface to the Levenberg-Marquardt Nonlinear Least-Squares Algorithm Found in MINPACK, Plus Support for Bounds. https://CRAN.R-project.org/package=minpack.lm

[CR20] Fenn WO (1930a) Frictional and kinetic factors in the work of sprint running. Am J Physiol 92(3):583–611. 10.1152/ajplegacy.1930.92.3.583

[CR21] Fenn WO (1930b) Work against gravity and work due to velocity changes in running: movements of the center of gravity within the body and foot pressure on the ground. Am J Physiol 93(2):433–462. 10.1152/ajplegacy.1930.93.2.433

[CR22] Fox EL, Kirkendall DT, Bartels RL (1980) Linear determination of the VO2 half time response during exercise. Eur J Appl Physiol 44(1):77–81. 10.1007/bf00421766 10.1007/BF00421766 7190499

[CR23] Francescato MP, Cettolo V, di Prampero PE (2003) Relationships between mechanical power, O(2) consumption, O(2) deficit and high-energy phosphates during calf exercise in humans. Pflugers Arch 445(5):622–628. 10.1007/s00424-002-0992-9 12634935 10.1007/s00424-002-0992-9

[CR24] Francescato MP, Cettolo V, di Prampero E (2008) Influence of phosphagen concentration on phosphocreatine breakdown kinetics. data from human gastrocnemius muscle. J Appl Physiol 105(1):158–164. 10.1152/japplphysiol.00007.2008 18436701 10.1152/japplphysiol.00007.2008

[CR25] Gastin PB (2001) Energy system interaction and relative contribution during maximal exercise. Sports Med 31(10):725–741. 10.2165/00007256-200131100-00003 11547894 10.2165/00007256-200131100-00003

[CR26] Gastin PB, Costill DL, Lawson DL, Krzeminski K, McConell GK (1995) Accumulated oxygen deficit during supramaximal all-out and constant intensity exercise. Med Sci Sports Exerc 27(2):255–263. 10.1249/00005768-199502000-00016 7723650

[CR27] Girard O, Mendez-Villanueva A, Bishop D (2011) Repeated-sprint ability - part I: factors contributing to fatigue. Sports Med 41(8):673–694. 10.2165/11590550-000000000-00000 21780851 10.2165/11590550-000000000-00000

[CR28] Gollnick PD, Hermansen L (1973) Biochemical adaptations to exercise: anaerobic metabolism. Exerc Sport Sci Rev 1:1–43 4806373

[CR29] Graubner R, Nixdorf E (2011) Biomechanical analysis of the sprint and hurdles events at the 2009 IAAF world championships in athletics. New Stud Athl 1(2):19–53

[CR30] Hanon C, Lepretre P-M, Bishop D, Thomas C (2010) Oxygen uptake and blood metabolic responses to a 400-m run. Eur J Appl Physiol 109(2):233–240. 10.1007/s00421-009-1339-4 20063105 10.1007/s00421-009-1339-4

[CR31] Hargreaves M, Spriet LL (2020) Skeletal muscle energy metabolism during exercise. Nat Metab 2(9):817–828. 10.1038/s42255-020-0251-4 32747792 10.1038/s42255-020-0251-4

[CR32] Harman C (2002) A biomechanical power model for world-class 400 metre running. Proceedings of Sixth Australian Conference on Mathematics and Computers in Sport. de Mestre, N, Ed. Queensland, Australia: Bond University, 155–166. http://www.mathsportinternational.com/anziam/Mathsport%206.pdf#page=164

[CR33] Hautier CA, Wouassi D, Arsac LM, Bitanga E, Thiriet P, Lacour JR (1994) Relationships between postcompetition blood lactate concentration and average running velocity over 100-m and 200-m races. Eur J Appl Physiol 68(6):508–513. 10.1007/bf00599521 10.1007/BF00599521 7957143

[CR34] Heck H, Schulz H, Bartmus U (2003) Diagnostics of anaerobic power and capacity. Eur J Sport Sci 3(3):1–23. 10.1080/17461390300073302

[CR35] Hermansen L (1981) Muscular fatigue during maximal exercise of sport duration. Physiological Chemistry of Exer. and Training. https://cir.nii.ac.jp/crid/1570854175184650368

[CR36] Hernández Gomez J, Marquina V, Gómez RW (2013) On the performance of usain bolt in the 100 m sprint. Eur J Phys 34(5):1227–1233. 10.1088/0143-0807/34/5/1227

[CR37] Hill AV (1925) The physiological basis of athletic records. Sci Monthly 21(4):409–428

[CR38] Hirvonen J, Rehunen S, Rusko H, Härkönen M (1987) Breakdown of high-energy phosphate compounds and lactate accumulation during short supramaximal exercise. Eur J Appl Physiol 56(3):253–259. 10.1007/bf00690889 10.1007/BF00690889 3569234

[CR39] Hirvonen J, Nummela A, Rusko H, Rehunen S, Härkönen M (1992) Fatigue and changes of ATP, creatine phosphate, and lactate during the 400-m sprint. Can J Sport Sci 17(2):141–144 1324108

[CR40] Hunter DW (2017) Race and athletic performance: a physiological review. Afr Am Sports. 10.4324/9781315082950

[CR41] Janssen I, Heymsfield SB, Wang ZM, Ross R (2000) Skeletal muscle mass and distribution in 468 men and women aged 18–88 yr. J Appl Physiol 89(1):81–88. 10.1152/jappl.2000.89.1.81 10904038 10.1152/jappl.2000.89.1.81

[CR42] Kennelly AE (1926) Changes during the last twenty years in the world’s speed records of racing animals. Proceed Am Acad Arts Sci 61(11):487–523. 10.2307/20026170

[CR43] Kersting UG (1997) Biomechanical analysis of the sprinting events. Biomechanical Research Project Athens

[CR44] Kindermann W (1977) Lactate acidosis with different forms of sports activities. Can J Appl Sports Sci 2:177–82

[CR45] Korhonen MT, Mero A, Suominen H (2003) Age-related differences in 100-m sprint performance in male and female master runners. Med Sci Sports Exerc 35(8):1419–1428. 10.1249/01.Mss.0000079080.15333.Ca 12900699 10.1249/01.MSS.0000079080.15333.CA

[CR46] Lacour JR, Bouvat E, Barthelemy JC (1990) Relationships between postcompetition blood lactate concentrations as indicators of anaerobic energy-expenditure during 400-m and 800-m races. Eur J Appl Physiol 61(3–4):172–176. 10.1007/bf00357594 10.1007/BF00357594 2282899

[CR47] Legaz-Arrese A, Munguía-Izquierdo D, Nuviala Nuviala A, Serveto-Galindo O, Moliner Urdiales D, Reverter Masía J (2007) Average VO2max as a function of running performances on different distances. Sci Sports 22(1):43–49. 10.1016/j.scispo.2006.01.008

[CR48] Limpert E, Stahel WA, Abbt M (2001) Log-normal distributions across the sciences: keys and clues: on the charms of statistics, and how mechanical models resembling gambling machines offer a link to a handy way to characterize log-normal distributions, which can provide deeper insight into variability and probability—normal or log-normal: That is the question. Bioscience 51(5):341–352

[CR49] Lloyd BB (1967) World running records as maximal performances. Circulation Res XX 21:218–226

[CR50] Mader A (2003) Glycolysis and oxidative phosphorylation as a function of cytosolic phosphorylation state and power output of the muscle cell. Eur J Appl Physiol 88(4–5):317–338. 10.1007/s00421-002-0676-3 12527960 10.1007/s00421-002-0676-3

[CR51] Margaria R (1938). Sulla fisiologia e specialmente sul consumo energetico della marcia e della corsa a varie velocita ed inclinazioni del terreno

[CR52] Medbo JI, Mohn AC, Tabata I, Bahr R, Vaage O, Sejersted OM (1988) Anaerobic capacity determined by maximal accumulated O2 deficit. J Appl Physiol 64(1):50–60. 10.1152/jappl.1988.64.1.50 3356666 10.1152/jappl.1988.64.1.50

[CR53] Mero A, Komi PV, Gregor RJ (1992) Biomechanics of sprint running: a review. Sports Med (Auckland, N.Z.) 13(6):376–392. 10.2165/00007256-199213060-00002 10.2165/00007256-199213060-00002 1615256

[CR54] Miller R, Balshaw TG, Massey GJ, Maeo S, Lanza MB, Haug B, Johnston M, Allen SJ, Folland JP (2024) Sex differences in muscle morphology between male and female sprinters. J Appl Physiol 136(6):1568–1579. 10.1152/japplphysiol.00009.2023 38660724 10.1152/japplphysiol.00009.2023 PMC11365543

[CR55] Minetti AE, Pavei G (2018) Update and extension of the “equivalent slope” of speed-changing level locomotion in humans: a computational model for shuttle running. J Exp Biol 221(Pt 15):jeb182303. 10.1242/jeb.182303 29895678 10.1242/jeb.182303

[CR56] Minetti AE, Moia C, Roi GS, Susta D, Ferretti G (2002) Energy cost of walking and running at extreme uphill and downhill slopes. J Appl Physiol 93(3):1039–1046. 10.1152/japplphysiol.01177.2001 12183501 10.1152/japplphysiol.01177.2001

[CR57] Morin J-B, Samozino P, Murata M, Cross MR, Nagahara R (2019) A simple method for computing sprint acceleration kinetics from running velocity data: Replication study with improved design. J Biomech 94:82–87. 10.1016/j.jbiomech.2019.07.020 31376978 10.1016/j.jbiomech.2019.07.020

[CR58] Moritani T, Nagata A, deVries HA, Muro M (1981) Critical power as a measure of physical work capacity and anaerobic threshold. Ergonomics 24(5):339–350. 10.1080/00140138108924856 7262059 10.1080/00140138108924856

[CR59] Morton RH (2006) The critical power and related whole-body bioenergetic models. Eur J Appl Physiol 96(4):339–354. 10.1007/s00421-005-0088-2 16284785 10.1007/s00421-005-0088-2

[CR60] Müller H, Hommel H (1997) Biomechanical Research Project at the VIth World Championships in Athletics, Athens 1997: Preliminary Report. New Studies in Athletics

[CR61] Murase Y, Hoshikawa T, Yasuda N, Ikegami Y, Matsui H (1976). Analysis of the changes in progressive speed during 100-meter dash

[CR62] Osgnach C, di Prampero PE, Zamparo P, Morin J-B, Pavei G (2023) Mechanical and metabolic power in accelerated running-Part II: team sports. Eur J Appl Physiol. 10.1007/s00421-023-05286-1 37535141 10.1007/s00421-023-05286-1

[CR63] Perez-Gomez J, Rodriguez GV, Ara I, Olmedillas H, Chavarren J, González-Henriquez JJ, Dorado C, Calbet JAL (2008) Role of muscle mass on sprint performance: gender differences? Eur J Appl Physiol 102(6):685–694. 10.1007/s00421-007-0648-8 18084774 10.1007/s00421-007-0648-8

[CR64] Péronnet F, Thibault G (1989) Mathematical analysis of running performance and world running records. J Appl Physiol 67(1):453–465. 10.1152/jappl.1989.67.1.453 2759974 10.1152/jappl.1989.67.1.453

[CR65] Plamondon A, Roy B (1984) Cinématique et cinétique de la course accélérée. Can J Appl Sport Sci 9(1):42–52 6705128

[CR66] Pugh LG (1970) Oxygen intake in track and treadmill running with observations on the effect of air resistance. J Physiol 207(3):823–835. 10.1113/jphysiol.1970.sp009097 5532903 10.1113/jphysiol.1970.sp009097 PMC1348744

[CR67] Rodríguez FA, Mader A (2011) Energy systems in swimming. World book of swimming: from science to performance, 225–240

[CR68] Saltin B, Astrand PO (1967) Maximal oxygen uptake in athletes. J Appl Physiol 23(3):353–358. 10.1152/jappl.1967.23.3.353 6047957 10.1152/jappl.1967.23.3.353

[CR69] Saltin B, Essén B (1971) Muscle glycogen, lactate, ATP, and CP in intermittent exercise. Advances in Experimental Medicine and Biology. Springer US, Boston, MA, pp 419–424

[CR70] Skiba PF, Chidnok W, Vanhatalo A, Jones AM (2012) Modeling the expenditure and reconstitution of work capacity above critical power. Med Sci Sports Exerc 44(8):1526–1532. 10.1249/MSS.0b013e3182517a80 22382171 10.1249/MSS.0b013e3182517a80

[CR71] Skiba PF, Fulford J, Clarke DC, Vanhatalo A, Jones AM (2015) Intramuscular determinants of the ability to recover work capacity above critical power. Eur J Appl Physiol 115(4):703–713. 10.1007/s00421-014-3050-3 25425258 10.1007/s00421-014-3050-3

[CR72] Stachoń A, Pietraszewska J, Burdukiewicz A (2023) Anthropometric profiles and body composition of male runners at different distances. Sci Rep. 10.1038/s41598-023-45064-9 37880292 10.1038/s41598-023-45064-9 PMC10600198

[CR73] Stryer L (1995) Biochemistry (4th ed., p. xxxiv + 1064). W.H. Freeman

[CR74] Taylor AW, Peronnet F (1981) Caracteristiques des fibres musculaires et lactacidémie à l’exercise chez l’homme et chez la femme avant et après entraînement. Med Sport 55:48–52

[CR75] Thongsit N, Sangnok Y, Wannapak W, Khoomsab K, Iamsamang S, Sangnok S, Hutem A (2019) Theory mathematics and physics model fitting of kinematic parameter for usain bolt 100 metres sprint at Beijing Olympic Games 2008. Appl Mech Mater 886:201–205

[CR76] Wang Z, Deurenberg P, Wang W, Pietrobelli A, Baumgartner RN, Heymsfield SB (1999) Hydration of fat-free body mass: review and critique of a classic body-composition constant. Am J Clin Nutr 69(5):833–841. 10.1093/ajcn/69.5.833 10232621 10.1093/ajcn/69.5.833

[CR77] Ward-Smith AJ (1985) A mathematical theory of running, based on the first law of thermodynamics, and its application to the performance of world-class athletes. J Biomech 18(5):337–349. 10.1016/0021-9290(85)90289-1 4008504 10.1016/0021-9290(85)90289-1

[CR78] Ward-Smith AJ (1999) Aerobic and anaerobic energy conversion during high-intensity exercise. Med Sci Sports Exerc 31(12):1855–1860. 10.1097/00005768-199912000-00025 10613440 10.1097/00005768-199912000-00025

[CR79] Ward-Smith AJ, Radford PF (2000) Investigation of the kinetics of anaerobic metabolism by analysis of the performance of elite sprinters. J Biomech 33(8):997–1004. 10.1016/s0021-9290(00)00035-x 10828330 10.1016/s0021-9290(00)00035-x

[CR80] Weigend FC, Siegler J, Obst O (2021) A new pathway to approximate energy expenditure and recovery of an athlete. In arXiv [cs.NE] (pp. 325–326). arXiv. 10.1145/3449726.3459469

