# Supplementary material for: Quantifying metabolic energy contributions in sprint running: a novel bioenergetic model
Source: Eur J Appl Physiol. 2025 Jun 19;125(12):3521–41. doi: 10.1007/s00421-025-05831-0 (PMC12678604; doi:10.1007/s00421-025-05831-0)
Supplement: Supplementary file 1 — Supplementary file1 (DOCX 35 KB) [file 421_2025_5831_MOESM1_ESM.docx]

**Supplementary Material**

**Video Footage of the Berlin 2009 Women’s 400 m**

Video analysis of the Women's 400 m race reveals that Richards (lane 3) appears to have executed a strategic acceleration early in the race, potentially to use Williams' slipstream (lane 4) in the backstretch. This could explain the observed slowdown in Richards second 100 m velocity profile.

<https://youtube.com/watch?v=VUIcvYnpHLQ>

**Error Analysis of the Instantaneous Velocity Model**

Table 1 displays the residual standard error (RSE) and root mean squared error (RMSE) values for the instantaneous velocity model, compared to the velocities reported by Graubner and Nixdorf (2011) for each sprint running event.

Table 1: Residual standard error (RSE) and root mean squared error (RMSE) of the velocity time course model applied to velocity data provided by Graubner and Nixdorf (2011) for all sprint races.

| Distance (m) | $\boldsymbol{RSE}$ (m s^-1^) | $\boldsymbol{RMSE}$ (m s^-1^) |
| --- | --- | --- |
| Men | | |
| 100 | 0.12 | 0.13 |
| 200 | 0.23 | 0.31 |
| 400 | 0.07 | 0.09 |
| Women | | |
| 100 | 0.14 | 0.18 |
| 200 | 0.30 | 0.39 |
| 400 | 0.17 | 0.22 |

**Derivation of the Bi-Exponential Model Normalization Constant**

We aim to normalize the function:

$$f\left( t \right)=\left( 1-e^{-t/t_{1}} \right)\cdot e^{-t/t_{2}}$$

such that its maximum value equals 1.

Step 1: Locate the Maximum

To find the maximum of f(t), compute the derivative $f^{'}\left( t \right)$ and solve $f^{'}\left( t \right)$= 0. Using the product rule:

$$f^{'}\left( t \right)=\frac{d}{dt}\left[ \left( 1-e^{-t/t_{1}} \right)\cdot e^{-t/t_{2}} \right]$$

The derivative expands as:

$$f^{'}\left( t \right)=\frac{d}{dt}\left( 1-e^{-t/t_{1}} \right)\cdot e^{-t/t_{2}}+\left( 1-e^{-t/t_{1}} \right)\cdot\frac{d}{dt}\left( e^{-t/t_{2}} \right)$$

Derivative of $1-e^{-t/t_{1}}$:

$$\frac{d}{dt}\left( 1-e^{-t/t_{1}} \right)=\frac{1}{t_{1}}e^{-t/t_{1}}$$

Derivative of $e^{-t/t_{2}}$:

$$\frac{d}{dt}\left( e^{-t/t_{2}} \right)=-\frac{1}{t_{2}}e^{-t/t_{2}}$$

Substitute these into $f^{'}\left( t \right)$:

$$f^{'}\left( t \right)=\frac{1}{t_{1}}e^{-t/t_{1}}\cdot e^{-t/t_{2}}-\frac{1}{t_{2}}\left( 1-e^{-t/t_{1}} \right)\cdot e^{-t/t_{2}}$$

Factor out $e^{-t/t_{2}}$:

$$f^{'}\left( t \right)=e^{-t/t_{2}}\left[ \frac{1}{t_{1}}e^{-t/t_{1}}-\frac{1}{t_{2}}+\frac{1}{t_{2}}e^{-t/t_{1}} \right]$$

Simplify:

$$f^{'}\left( t \right)=e^{-t/t_{2}}\left[ \left( \frac{1}{t_{1}}+\frac{1}{t_{2}} \right)e^{-t/t_{1}}-\frac{1}{t_{2}} \right]$$

Set $f^{'}\left( t \right)$ = 0:

$$\left( \frac{1}{t_{1}}+\frac{1}{t_{2}} \right)e^{-t/t_{1}}=\frac{1}{t_{2}}$$

Solve for $e^{-t/t_{1}}$:

$$e^{-t/t_{1}}=\frac{\frac{1}{t_{2}}}{\frac{1}{t_{1}}+\frac{1}{t_{2}}}=\frac{t_{1}}{t_{1}+t_{2}}$$

Take the natural logarithm to find the critical point $t_{\text{max}}$:

$$t_{\text{max}}=-t_{1}\ln\left( \frac{t_{1}}{t_{1}+t_{2}} \right)$$

Step 2: Compute the Maximum Value

Substitute $t_{\text{max}}$into f(t):

$$f\left( t_{\text{max}} \right)=\left( 1-e^{-t_{\text{max}}/t_{1}} \right)\cdot e^{-t_{\text{max}}/t_{2}}$$

Compute $1-e^{-t_{\text{max}}/t_{1}}$:

$$e^{-t_{\text{max}}/t_{1}}=\frac{t_{1}}{t_{1}+t_{2}}$$

so:

$$1-e^{-t_{\text{max}}/t_{1}}=1-\frac{t_{1}}{t_{1}+t_{2}}=\frac{t_{2}}{t_{1}+t_{2}}$$

Compute $e^{-t_{\text{max}}/t_{2}}$: From $t_{\text{max}}=-t_{1}\ln\left( \frac{t_{1}}{t_{1}+t_{2}} \right)$:

$$e^{-t_{\text{max}}/t_{2}}=e^{\frac{t_{1}}{t_{2}}\ln\left( \frac{t_{1}}{t_{1}+t_{2}} \right)}=\left( \frac{t_{1}}{t_{1}+t_{2}} \right)^{t_{1}/t_{2}}$$

Combine the two terms:

$$f\left( t_{\text{max}} \right)=\frac{t_{2}}{t_{1}+t_{2}}\cdot\left( \frac{t_{1}}{t_{1}+t_{2}} \right)^{t_{1}/t_{2}}$$

Let $f_{\text{max}}=f\left( t_{\text{max}} \right)$:

$$f_{\text{max}}=\frac{t_{2}}{t_{1}+t_{2}}\cdot\left( \frac{t_{1}}{t_{1}+t_{2}} \right)^{t_{1}/t_{2}}$$

Step 3: Normalize the Function

To normalize f(t), divide it by $f_{\text{max}}:$

$$f_{\text{normalized}}\left( t \right)=\frac{f\left( t \right)}{f_{\text{max}}}$$

Substitute f(t) and $f_{\text{max}}:$

$$f_{\text{normalized}}\left( t \right)=\frac{\left( 1-e^{-t/t_{1}} \right)\cdot e^{-t/t_{2}}}{\frac{t_{2}}{t_{1}+t_{2}}\cdot\left( \frac{t_{1}}{t_{1}+t_{2}} \right)^{t_{1}/t_{2}}}$$

Simplify:

$$f_{\text{normalized}}\left( t \right)=\frac{\left( 1-e^{-t/t_{1}} \right)\cdot e^{-t/t_{2}}\cdot\left( t_{1}+t_{2} \right)}{t_{2}\cdot\left( \frac{t_{1}}{t_{1}+t_{2}} \right)^{t_{1}/t_{2}}}$$

Final Normalized Function

The normalized function is:

$$f_{\text{normalized}}\left( t \right)=\frac{\left( 1-e^{-t/t_{1}} \right)\cdot e^{-t/t_{2}}\cdot\left( t_{1}+t_{2} \right)}{t_{2}\cdot\left( \frac{t_{1}}{t_{1}+t_{2}} \right)^{t_{1}/t_{2}}}$$

This ensures max($f_{\text{normalized}}\left( t \right)$) = 1.

**Initial Parametrization of the Sprint Bioenergetic Model**

We calculated approximate metabolic contributions for aerobic, lactic, and alactic systems in the female 100 m sprint, and the male and female 200 m and 400 m sprints. This approximation used the methodology outlined in the main text for the male 100 m sprint (Figure 3). We applied Equations 5 and 6 to the approximate contributions to establish initial model parameters and assess consistency. Table 6 summarizes this parameterization. Parameters were obtained from a non-linear least-squares fit on approximate contributions of aerobic, lactic and alactic energy systems from World record performance metabolic power data.

Following the initial parameterization, we formalized a general model for male and female 100, 200, and 400 m sprints. We fixed parameters $k_{1}$, $k_{2}$, $\mu_{al}$, and $\sigma_{al}$ for parsimony and simplicity. Thus, variations in $P_{la,max}\left( T \right)$, $P_{al,max}\left( T \right)$, and MAP alone shape the model’s time course over sprint duration T. Here, we set MAP at 24.5 W kg^-1^ for men and 21 W kg^-1^ for women, simplifying the analysis. Table 2 (below) details the fixed values for $k_{1}$, $k_{2}$, $\mu_{al}$, and $\sigma_{al}$ in the formalized model.

*Table 2 : Initial parameterization via non-linear least-squares fit. We aligned the alactic and lactic models with approximate metabolic power distributions of each energy system in male and female 100, 200, and 400 m sprints. The final column shows the formalized model’s parametrization, with only* $P_{la,max}\left( T \right)$ *and* $P_{al,max}\left( T \right)$ *as variable parameters. Fixed parameters* $\mu_{al}$*, and* $\sigma_{al}$ *set maximum alactic power to peak at ~0.5 s. Its contribution diminishes rapidly, vanishing by ~15 s. The values of* $k_{1}$ *and* $k_{2}$ *align with theoretical considerations from the main text.*

| Parameter | Men’s  100 m | Men’s  200 m | Men’s  400 m | Women’s  100 m | Women’s  200 m | Women’s  400 m | Formalized Model |
| --- | --- | --- | --- | --- | --- | --- | --- |
| $\boldsymbol{P}_{\boldsymbol{al}\boldsymbol{,}\boldsymbol{max}}$ (W kg^-1^) | 144 | 83 | 60 | 89 | 62 | 51 | *Variable* |
| $\boldsymbol{\sigma}_{\boldsymbol{al}}$ | 0.98 | 0.98 | 0.99 | 1.01 | 0.99 | 1.01 | 1 |
| $\boldsymbol{\mu}_{\boldsymbol{al}}$ | -0.61 | -0.35 | -0.32 | -0.51 | -0.31 | -0.31 | -0.4 |
| $\boldsymbol{P}_{\boldsymbol{la}\boldsymbol{,}\boldsymbol{max}}$ (W kg^-1^) | 57 | 51 | 38 | 44 | 42 | 35 | *Variable* |
| $\boldsymbol{k}_{\boldsymbol{1}}$ (s) | 3.02 | 2.56 | 2.45 | 2.93 | 2.84 | 2.64 | 2.75 |
| $\boldsymbol{k}_{\boldsymbol{2}}$ (s) | 21.59 | 33.99 | 42.43 | 26.86 | 31.25 | 42.09 | 35 |

**Formulation of the Bioenergetic Model of Sprint Running**

Complete formulation of the bioenergetic model of sprint running.

$$P_{tot}\left( t \right)= P_{al}\left( t \right)+ P_{la}\left( t \right)+ P_{aer}\left( t \right)$$

$$P_{tot}\left( t \right) =P_{al,max}\left( T \right){\cdot e}^{\left( \frac{{-\left( \ln\left( t \right)- \mu_{al} \right)}^{2}}{{2\sigma_{al}}^{2}} \right)} + P_{la,max}(T){\cdot k_{norm}\cdot(1-e}^{\frac{-t}{k_{1}}})\cdot e^{\frac{-t}{k_{2}}} + \left( MAP-BMR \right)\left( 1-e^{\frac{-t}{k_{aer}}} \right)$$

Where

$$P_{al,max}\left( T \right) = \frac{E_{al,max}}{T}{\cdot e}^{\left( \frac{{-\left( \ln\left( T \right)- 1.75 \right)}^{2}}{2\left( 1.5 \right)^{2}} \right)} \left( \int_{0}^{T} e^{\left( \frac{{-\left( \ln\left( t \right)- \mu_{al} \right)}^{2}}{{2\sigma_{al}}^{2}} \right)} dt \right)^{-1}$$

And

$$P_{la,max}\left( T \right) = E_{la,max}{\cdot k_{norm,2}\cdot(1-e}^{\frac{-\left( T - 3 \right)}{20}})\cdot e^{\frac{-\left( T-3 \right)}{1500}} \left( \int_{0}^{T} {\cdot k_{norm}\cdot(1-e}^{\frac{-t}{k_{1}}})\cdot e^{\frac{-t}{k_{2}}} dt \right)^{-1}$$
